# Business grants following natural disasters and their different impact on the performance of female and male-owned microenterprises: Evidence from Sri Lanka

**DOI:** 10.1371/journal.pone.0279418

**Published:** 2022-12-21

**Authors:** Ha Luong

**Affiliations:** 1 Department of Economics, University of Barcelona, Barcelona, Spain; 2 Barcelona Institute of Economics (IEB), Barcelona, Spain; University of Almeria: Universidad de Almeria, SPAIN

## Abstract

**Objective:**

This paper investigates gender differences in the treatment effects of business grants on firm performance following natural disasters, and seeks to identify the mechanisms underlying the unequal effects.

**Method:**

A panel data-set from an experiment in Sri Lanka is used to measure the difference in the treatment effects of a business grant on the performance of female and male-owned firms following the 2004 Indian Ocean tsunami. The sample of 608 microenterprises includes 297 female-owned firms and 311 male-owned firms. There are 338 firms (Male = 176, Female = 162) in the treatment group that received the grant and 270 firms (Male = 135, Female = 135) in the control group that did not receive the grant. Data on firm performance, firm characteristics and owner characteristics were collected in 13 survey waves from April 2005 to December 2010. Firm performance, which is measured by firm profit, is assessed by employing linear regression with fixed effects in an intention-to-treat analysis.

**Findings:**

The results suggest that the business grant has a positive impact on the performance of male-owned firms, but zero effect on that of female-owned firms. Several potential mechanisms drive the results, including gender differences in business investment, household expenditure and initial business closures. The results also show a positive treatment effect of the business grant on the psychological recovery of recipients, but there is no evidence supporting gender differences in this dimension.

**Contribution:**

This paper provides new evidence on gender differences in the treatment effects of business grants on firm performance in the context of post-disasters, and has implications for business recovery programs aimed at supporting female microentrepreneurs in the aftermath of large-scale catastrophes.

## Introduction

Natural disasters have posed massive challenges for humans throughout history. On average, such extreme large-scale events kill 45,000 people per year and account for 0.1% of global deaths [1]. Moreover, natural disasters resulted in massive economic losses of approximately one trillion U.S. dollars from 1980 to 2004 [2]. Out of all the highly vulnerable groups, micro, small and medium-sized enterprises (MSMEs), which are arguably the backbone of economic growth, have been affected particularly severely by these catastrophic weather events. Owing to their limited preparedness and inadequate financial resources, the physical damage and lifeline disruption caused by natural disasters often lead to negative consequences on MSME operations. The Pakistan floods of 2010, for example, caused 75.4% of surviving firms to run at a loss compared to their situation before the floods, and only 7.7% were able to keep their operation at the same level [3]. Moreover, large-scale catastrophes can force MSMEs to close down or relocate in their aftermath [4]. For instance, around 43% of U.S. enterprises that have experienced disasters have never reopened, and another 29% of them have closed within two years [5].

With regard to the gender of owners, the disaster literature suggests that female owners are more likely than their male counterparts to face greater obstacles. In the context of the United States, female-led enterprises are more likely to be rejected than male-led enterprises when they apply for Small Business Administration (SBA) disaster loans [6]. In addition, natural disasters tend to reinforce the disadvantages of female owners compared to male owners when they begin their businesses. For example, they have lower capital and less insurance, which in turn leads to slower recovery and lower rates of survival [7]. In the wake of natural disasters, a common way to support MSMEs is to provide business grants to remove credit constraints, assist in the recovery process and strengthen resilience. A few papers report a positive impact of business grants on firm survival [8] and on firm revenue, profits and savings [9]. However, there is little evidence about gender differences in relation to the impact of business grants on firm performance following extreme weather events.

Given the gap in knowledge, this paper aims to investigate whether female-owned and male-owned microenterprises reap the same benefit from a business grant following the 2004 Indian Ocean tsunami in Sri Lanka. Sri Lanka provides an interesting context for study. First, the country was completely unprepared for the tsunami since the previous tsunami generated by the Krakatoa volcanic eruption, which happened in 1883, had little effect on Sri Lanka. By contrast, the 2004 Indian Ocean tsunami is considered to be one of the deadliest natural disasters in the country, resulting in over 36,000 deaths and the displacement of 800,000 people (Department of Census and Statistics, 2005). Second, MSMEs are the foundation of the Sri Lankan economy, contribute 52% to the country’s gross domestic product (GDP) and account for 90% of all businesses. Following the tsunami, it is estimated that around 25,000 microenterprises were damaged by the large-scale disaster. Third, despite some significant progress on women’s rights, gender inequality is still a prevalent issue in Sri Lanka. Sri Lankan women face several persistent challenges such as limited education, intimate partner violence and traditional gender roles around family responsibilities. Moreover, the post-tsunami period created more challenges for women in the country since they faced economic hardship and increased care-giving stress and burnout [10].

To shed light on the issue, this study uses a panel dataset from an experiment that was conducted in Sri Lanka after the 2004 Indian Ocean tsunami. Carried out by researchers from the World Bank Group, Sri Lanka’s University of Peradeniya and the United Kingdom’s University of Warwick [11], the experiment focused on low-capital microenterprises in three coastal districts that were severely affected by the tsunami. A baseline survey was conducted in April 2005 after a screening survey of households from 25 divisions in the 2001 Sri Lankan census. Following the baseline survey, the research group randomly assigned one-time grants of either 10,000 or 20,000 Sri Lanka rupees (LKR), which was equivalent to 100 U.S. dollars or 200 U.S. dollars (exchange rate in 2005) in the form of cash or in-kind payments to enterprises. Researchers interviewed enterprises in 11 waves from April 2005 to April 2008 and then 2 follow-up waves in 2010. This study mainly focuses on the first 11 waves to measure the short-term impact of the business grant. The results suggest that the business grant has different effects on the performance of male-owned firms and female-owned firms. The potential underlying mechanisms include gender differences in business investment, household expenditure and initial business closures. Moreover, the grant has a positive effect on the psychological recovery of small business owners. Note that even though gender refers to the socially constructed characteristics of women and men while sex refers to the different biological and physiological characteristics of males and females, in this paper, gender and sex are used as synonyms.

There are three related research articles that utilize data from this experiment. De Mel et al. [12] and De Mel et al. [13] examine the return to capital and the gender gap in the treatment effect among a sub-sample of indirectly affected and unaffected firms, since they argue that the recovery process of directly affected firms might have an impact on the return to capital. More specifically, the authors in De Mel et al. [12] explore the effects on firm performance of four treatment types, which are classified by method (cash or in-kind) and amount (10,000 or 20,000 LKR). Their results suggest that all treatments (10,000 LKR cash, 10,000 LKR in-kind, 20,000 LKR cash, 20,000 LKR in-kind) have positive impacts on capital stock, while three treatments have positive effects on firm profit. In addition, they show that the real return to capital in the experiment is higher than the market interest rate. They then provide evidence of heterogeneous treatment effects across the characteristics of owners and the household of owners. Their results indicate that the high real return to capital is likely to reflect credit constraints among microenterprises.

In De Mel et al. [13], the authors investigate the mean treatment effects and returns to capital by gender. They find a large positive and significant effect for male-owned firms, but zero effect for female-owned firms. Subsequently, they offer some potential mechanisms, including gender differences in investing the grant in capital stock, the association between behaviors of female recipients and whether they were in male or female-dominated sectors, and the evidence that grants to women were “captured” by their spouses. The same group of authors explore enterprise recovery following a natural disaster, but without the gender perspective in De Mel et al. [14]. In order to estimate the treatment effects on capital stock, business income and enterprise recovery, they compare firms in directly affected zones with firms in indirectly affected and unaffected zones. Their key findings are that business grants speed up the recovery of small enterprises, and that firms in retail sectors benefit more from the grants than firms in manufacturing and service sectors.

The present study differs from the aforementioned papers in the following ways: (i) the study treats all firms in the experiment as if they experienced some potential impacts of the tsunami, but not uniformly so; (ii) it provides new insights into possible mechanisms that are closely related to the recovery process, including household expenditure on basic needs and initial business closures; (iii) it explores the treatment effect and potential gender differences in the psychological recovery of owners following the tsunami; and (iv) the external validity of the main results is tested using microenterprise level data in the context of coronavirus disease (COVID-19) pandemic.

First, the results suggest that the business grant has a significantly positive impact on the performance of male-owned firms, but zero effect on that of female owned-firms. On average, male-owned firms have a 1,878 LKR increase in profit, which is 41.5% of the baseline profit of the control group. The profit of female-owned firms is around 1,552 LKR smaller than their male counterparts. The overall female treatment effect on profit is then tested, and the results indicate that the null hypothesis of zero treatment effect on the performance of female-owned firms is not rejected. The baseline result holds when other observable characteristics that might differ between female and male owners besides their gender are added to the regression model. In addition, treatment effects are heterogeneous across treatment amounts, sectors and levels of asset damage caused by the tsunami.

Next, the study explores the underlying mechanisms that drive the results. The heterogeneous treatment effects within the subgroups receiving 10,000 LKR and 20,000 LKR suggest that the two treatment amounts should be analyzed separately. The findings suggest that female owners in the subgroup receiving the smaller treatment amount invest less in capital stock and inputs, but pay more interest on firm loans than their male counterparts. In the subgroup receiving the higher treatment amount, female owners spend more on their household basic needs than male owners. In addition, the paper investigates gender differences in closing the initial business. The results indicate that female owners in the treatment group, especially in the subgroup receiving 10,000 LKR, are more likely to change the line of their business or change both the location and line of their business, or no longer be engaged in self-employment, than other subgroups.

Further, the paper presents evidence of treatment effects on the psychological recovery of small business owners following natural disasters. The finding suggests that receiving a business grant has a significant and positive impact on recipients’ mental recovery. However, there is no evidence supporting gender differences in this dimension.

Finally, the external validity of the main results is confirmed in the context of the COVID-19 pandemic. The findings indicate that the sales of female-owned firms decrease significantly more than those of male-owned firms even though they receive similar business supports. This result provides suggestive evidence that the main results appear to hold in other contexts, and are not limited to Sri Lanka.

## Literature review

There are two main doctrines related to the literature concerned with gender differences in firm performance: liberal feminism and social feminism [15]. Liberal feminist theory explains that outcomes are different between men and women because women face discrimination that prevents them from accessing vital resources [16]. If women and men are given equal opportunities, women and men will attain their capacities more equally, and the observed differences in outcomes will then diminish. On the contrary, social feminist theory holds the belief that men and women are not the same by nature, and these differences (such as traits and/or experiences) may cause them to have different behaviors that affect firm performance. In the situation that female-owned firms and male-owned firms receive the same business intervention, liberal feminism theory suggests that they should benefit equally from the intervention. Similarly, despite men and women being inherently different by nature, social feminist theory does not imply any differences in their firm performance based on these characteristics. Therefore, female-owned firms and male-owned firms should reap similar benefits from the same business intervention under both theories.

In most experimental studies, however, the empirical literature documents that gender differences do exist in relation to the return on business interventions under normal circumstances. For instance, Gine and Mansuri [17] find that a business training program in rural Pakistan has a positive effect only on the business outcomes of small male businesses, not on those of their female counterparts. In the context of Ghana, Fafchamps et al. [18] report an increase in profit from an in-kind grant to both male and female-owned firms, but the effect on male-owned firms is larger. Moreover, only female-run businesses with high initial profit (the largest 40% of firms) benefit from the in-kind grant. With respect to a cash grant, they document some impacts on male businesses, but no impact on female businesses. A more recent work by Fiala [19] confirms gender differences in the effects of micro-credit in Uganda, finding that there is zero effect on the business outcomes of female-run businesses, but a positive and significant effect on those of male-run businesses. While previous studies focus solely on normal situations, this paper extends the literature by shedding light on gender differences in returns to a grant given in the context of post-disasters. More specifically, the study tests the following hypothesis: *An experimental business intervention following natural disasters has the same impact on the performance of female and male-owned microenterprises*.

In addition, the present study is connected to the literature that investigates the relationship between small business grants and firm survival. Previous studies investigate the grant effect on the survival probabilities of all firms without addressing the gender aspect of owners or by focusing only on female-owned firms. Overall, in both normal situations and recessions, small businesses tend to benefit from business grants through an improvement in their likelihood of survival. Pellegrin & Muccigrosso [20], for example, report the positive effect of capital subsidies on start-up survival in the south of Italy. In the context of Croatia, Srhoj et al. [21] document that entrepreneurship grants to women increase the chance of firm survival among both young and mature women entrepreneurs. The recent work of Srhoj et al. [22], also in Croatia, provides evidence of a positive effect from business development grants on young small firm survival during the recession from 2009 to 2014. The present paper contributes to this body of literature by exploring gender differences in initial business closures in the short term after receiving an experimental business grant following natural disasters. More specifically, small women-owned businesses are more likely than small men-owned businesses to close their firms.

Finally, this paper is relevant to the growing literature that examines the relationship between cash transfer programs and mental health outcomes. Previous studies focus mostly on children or households, and the results have been inconclusive. Several papers report a positive impact from cash transfers on the mental health outcomes of beneficiaries, including Haushofer and Shapiro [23], Kilburn et al. [24] and Shangani et al. [25] in Kenya, Angeles et al. [26] in Malawi, Ohrnberger et al. [27] in South Africa, and Tozan et al. [28] in Uganda. In contrast, some papers document zero effect of cash transfer programs on the psychological well-being of household members. For instance, Paxson & Shady [29] suggest no improvement in maternal mental health from the Bono de Desarrollo Humano program in Ecuador. Özler et al. [30] find a similar result on the effect of a gender mentoring and cash transfer intervention on girls aged 13 or 14 in Liberia. However, there is scarce evidence in the context of post-disasters and when small business owners are the targeted beneficiaries. This paper is the first attempt to experimentally examine the effect of receiving a business grant on the psychological recovery of microentrepreneurs.

## Materials and methods

The main data in the study come from an experiment that was conducted by researchers from the World Bank Group, the University of Peradeniya in Sri Lanka and the University of Warwick in the United Kingdom [11]. The investigators confirmed that Human Subjects approval for the study was obtained from the University of California, San Diego’s Human Research Protections Program, project number 061050S. The project’s title is “Rebuilding Sri Lankan Microenterprise After the Tsunami”. Information on the general purpose of the study was provided to participants and their written informed consent was obtained [31].

### Experiment

#### Experimental design and survey data

On December 26, 2004, a tsunami struck Sri Lanka in the wake of a magnitude 9.2 earthquake in Sumatra. This is considered to be the most devastating tsunami in Sri Lankan history. The total amount of physical damage was approximately 1.5 billion U.S. dollars, which was 7.2% of Sri Lanka’s GDP in 2004 [32]. The coastlines were severely affected, resulting in over 30,000 deaths, the displacement of thousands of households, and major losses of livelihood capital, community infrastructure, buildings and roads [33]. Following the tsunami, researchers from the World Bank Group, the University of Peradeniya in Sri Lanka and the University of Warwick in the United Kingdom designed a randomized control trial to quantify the impact of providing a business grant to microenterprises in three coastal southern and south-western districts (Kalutara, Galle, and Matara). The three districts were severely impacted with large numbers of deaths, missing people, displaced families, and losses of assets, poultry and livestock.

The target population of the experiment consisted of low-capital enterprises with less than 100,000 LKR in capital (excluding land and buildings), no paid employees, and owners who worked at least 30 hours per week and were from 20 to 65 years old. Using the 2001 Sri Lankan census, researchers selected 25 Grama Niladhari divisions (GNs) in Kalutara, Galle, and Matara. A GN is an administrative unit consisting of around 400 households on average. The choice was made based on a number of factors, including a high percentage of own-account workers and modest education levels, in order to yield a sufficient number of enterprises whose invested capital lay below the threshold. In the next step, researchers administered a screening survey of 3,361 households to identify firms with owners that met the experiment’s criteria. The baseline survey was then carried out with 659 firm owners in April 2005. After reviewing the baseline data, researchers dropped 42 firms whose capital exceeded the threshold, leaving 617 microenterprises in the baseline sample. However, the number of firms dropped from 617 to 608 between the first and second waves. Following [14], the present study focuses on the 608 firms that appeared in both waves. Microenterprises in the sample were classified into three groups, including firms in directly affected zones, firms in indirectly affected zones and firms in unaffected zones, which were located 410 meters (205 firms), 750 meters (208 firms) and 5.2 kilometers (195 firms) from the coast, respectively.

After the baseline survey, a one-time grant of either 10,000 or 20,000 LKR in the form of cash or business equipment was randomly provided to enterprises in May 2005 and November 2005. With respect to business equipment, first the enterprise owner chose the material, and then research staff from the project purchased the material for the firm. In the case of cash grants, the owners were told to purchase anything without restriction. In the experiment, the number of firms that received the business grant of 10,000 LKR and 20,000 LKR accounted for two-thirds and one-third of all treated firms, respectively. Note that the larger grant was approximately 80% of the median pre-tsunami capital stock of firms that suffered some damage from the tsunami.

At the first point of treatment (May 2005), the grant was received by 88 firms in directly affected zones, 72 firms in indirectly affected zones and 54 firms in unaffected zones, leaving 394 firms in the control group. At the second point of treatment (November 2005), out of the 394 firms that did not previously receive the business grant, 29 firms, 39 firms and 60 firms in directly affected, indirectly affected and unaffected zones, respectively, were provided cash or business equipment in one of two treatment amounts. From April 2006, the research team also initiated giving a token cash payment of 2,500 LKR to enterprises that had not received any treatment as an incentive to take part in the survey. In total, the survey includes thirteen waves of data collection. S1 Fig shows the timeline of the treatment and survey waves in detail. Moreover, in four waves of the panel, a complementary household survey was administered to gather information about the households of baseline owners.

While the experiment included firms in unaffected zones, the present study treats all enterprises as if they had experienced some potential impacts of the tsunami, though not uniformly so. This is because natural disasters often lead to broken supply chains [34], harder market conditions and shrinking demand for firms in affected regions owing to financial difficulties and the displacement of clients. The data from the baseline survey suggest that in the unaffected zones, approximately 32% of firms bought inputs from suppliers in the same divisional secretariats (DS are administrative sub-units of districts in Sri Lanka with a population of around 60,000) but different GNs, while 25% of firms bought inputs from suppliers in the same district but different DS. Moreover, 36% of those firms had customers in the same GN more than one kilometer from businesses, while 28% had customers in the same DS but a different GN. With respect to business demand, in the unaffected zones, 36% of firms reported that they had fewer customers in the baseline survey compared to their usual number of customers before the tsunami, and 14% of owners of those firms directly witnessed the tsunami. Further, 10% of firms in the indirectly affected zones had business assets damaged or destroyed by the tsunami. Therefore, the article argues that all firms in the experiment found themselves in a context of post-disaster recovery.

Attrition in data collection is quite low. In the baseline survey, there were 311 male-owned and 297 female-owned firms. Out of all the firms that reported their profits, 271 male-owned firms (90.6%) and 253 female-owned firms (89.7%) continued to provide information about profits and sales over the five waves. Moreover, the attrition rates in the female and male sub-samples are very similar, with 77.9% of 299 male-owned firms and 76.2% of 282 female-owned firms reporting their profits in all 11 waves.

#### Randomization check

The randomization was stratified by computer based on district (Kalutara, Gale and Matara) and level of damage caused by the tsunami (directly affected, indirectly affected and unaffected). Since gender is not a criterion in the randomization process, it is necessary to verify randomization in regard to the gender of owners.

Table 1 provides the balance test on observable characteristics in the male and female sub-samples. In terms of owner characteristics, on average, the treatment group and the control group in both sub-samples are very comparable, without any statistically significant differences. With respect to firm characteristics, on average, there are no significant differences between treated and control female-owned firms. In the male sub-sample, treated and control male-owned firms are different only by sector, which is represented by the variable *Retail/trade* that equals 1 if the firm is in the retail or trade sectors, and 0 when the firm belongs to the manufacturing or service sectors. On average, the treatment group has more male-owned firms in retail and trade sectors than the control group. The result suggests that even though the randomization is not stratified by gender, the treatment group and control group by gender are balanced on observable characteristics. Table A. in S1 File sets out the list and definition of variables used in the study.

**Table 1:** Randomization check by gender.

	Male			Female		
Variable	Control	Treat	P-val. diff	Control	Treat	P-val. diff
*Owner characteristics*						
Ability	-0.071	0.009	(0.578)	0.010	0.025	(0.923)
Experience	0.695	0.652	(0.440)	0.639	0.591	(0.414)
Age	42.370	42.528	(0.902)	41.030	41.741	(0.583)
Married	0.815	0.881	(0.106)	0.748	0.790	(0.393)
Migrant	0.148	0.091	(0.119)	0.119	0.148	(0.458)
Working hour	0.593	0.608	(0.785)	0.385	0.383	(0.965)
Household size	4.948	5.085	(0.493)	4.867	4.833	(0.872)
*Firm characteristics*						
Pre-investment	0.637	0.631	(0.909)	0.459	0.500	(0.486)
Retail/trade	0.407	0.506	(0.085)*	0.333	0.352	(0.739)
Firm age	10.170	12.108	(0.119)	10.176	10.578	(0.738)
Real profit	4,523	4,500	(0.961)	2,813	2,819	(0.986)
Real sales	14,036	14,259	(0.911)	8,802	8,541	(0.847)
Invested capital without land	32,472	30,394	(0.551)	21,524	24,355	(0.338)
Total number of workers	1.356	1.466	(0.133)	1.407	1.457	(0.497)
Interest payment	50.000	136.875	(0.178)	136.874	82.407	(0.426)
Observations	135	176		135	162	

*Note*:

*p < 0.10,

**p < 0.05,

***p < 0.01.

Table 2 further reports comparability within gender groups in two sub-samples of firms with and without tsunami-induced asset damage. In both sub-samples, there is no statistically significant difference, on average, between treated and control female-owned firms, except for owner ability in the sub-sample of firms with asset damage. With regard to male-owned firms, the treated group is weakly but significantly different from the control group in terms of owner ability, owner migration, firm sector (in the sub-sample of firms without asset damage) and total number of workers (in the sub-sample of firms with asset damage). Overall, even though the number of treated and control units in each category (classified by level of business asset damage and gender of owners) is not very large, the treatment and control groups are still quite comparable.

**Table 2:** Balance test by gender and by asset damage caused by the tsunami.

	With asset damage						Without asset damage					
	*Male*			*Female*			*Male*			*Female*		
Variable	Cont.	Treat	P-val.	Cont.	Treat	P-val.	Cont.	Treat	P-val.	Cont.	Treat	P-val.
*Owner characteristics*												
Ability	0.351	0.004	(0.167)	-0.061	0.363	(0.062)*	-0.308	0.012	(0.066)*	0.054	-0.206	(0.194)
Experience	0.778	0.627	(0.112)	0.698	0.689	(0.922)	0.651	0.664	(0.851)	0.608	0.523	(0.272)
Age	40.146	42.897	(0.199)	41.625	42.373	(0.720)	43.598	42.347	(0.438)	40.701	41.295	(0.722)
Married	0.833	0.914	(0.212)	0.813	0.791	(0.779)	0.805	0.864	(0.252)	0.713	0.789	(0.232)
Migrant	0.083	0.069	(0.783)	0.167	0.179	(0.864)	0.184	0.102	(0.091)*	0.092	0.126	(0.462)
Working hours	0.500	0.569	(0.483)	0.250	0.313	(0.463)	0.644	0.627	(0.809)	0.460	0.432	(0.704)
Household size	4.542	4.672	(0.675)	4.750	5.119	(0.310)	5.172	5.288	(0.648)	4.931	4.632	(0.227)
*Firm characteristics*												
Pre-investment	0.604	0.586	(0.853)	0.500	0.537	(0.696)	0.655	0.653	(0.969)	0.437	0.474	(0.620)
Retail/Trade	0.458	0.483	(0.804)	0.208	0.328	(0.159)	0.379	0.517	(0.051)*	0.402	0.368	(0.641)
Firm age	10.833	13.707	(0.160)	11.522	11.303	(0.915)	9.805	11.322	(0.333)	9.447	10.074	(0.675)
Real profit	4,619.768	4,237.719	(0.716)	2,304.889	2,613.594	(0.502)	4,475.000	4,632.301	(0.745)	3,089.819	2,966.000	(0.786)
Real sales	11,832.292	14,422.414	(0.482)	7,016.042	7,613.060	(0.737)	15,252.874	14,179.695	(0.650)	9,787.988	9,196.000	(0.755)
Invested capital without land	34,293.645	31,615.346	(0.722)	22,413.438	28,311.270	(0.263)	31,467.068	29,794.660	(0.642)	21,033.391	21,565.684	(0.879)
Total number of workers	1.417	1.638	(0.088)*	1.417	1.448	(0.816)	1.322	1.381	(0.500)	1.402	1.463	(0.473)
Interest payment	45.833	248.276	(0.198)	340.583	80.597	(0.119)	52.299	82.119	(0.584)	24.483	83.684	(0.122)
Observations	48	58		48	67		87	118		87	95	

*Note*:

*p < 0.10,

** *p* < 0.05,

***p < 0.01.

### Empirical model

The objective of the study is to measure the difference in treatment effects on the performance of male-owned firms and female-owned firms. The paper employs an empirical model that is close to the model of De Mel et al. [13]. However, the variable of interest in the study is *Treatment*, which is a binary variable and indicates whether a firm received a grant in a specific survey wave. In De Mel et al. [13], the authors use the treatment amount that was assigned to each enterprise as the variable of interest. Moreover, they include the 2,500 LKR payment as treatment, but they note that the result is unchanged if the payment is ignored. Therefore, there is a little concern in using a binary treatment variable. Provided that there are two treatment amounts (10,000 LKR and 20,000 LKR) and two methods of assigning treatment (cash and business equipment), it is important to test whether there is any difference in the average treatment effects across the four types of treatment. Table B. in S1 File indicates that there are no statistically significant differences in the average treatment effects on firm outcomes across all types of treatment. Therefore, the study proceeds with a binary treatment variable.

To capture the treatment impact on the performance of male-owned and female-owned firms, the paper uses a panel data model with firm fixed effects and wave fixed effects. Firm fixed effects are included in the model to control for any time-invariant characteristics of a firm that might affect firm performance. Wave fixed effects assume that the time paths of firm outcomes are the same for male-owned firms and female-owned firms. The specification of the model is as follows:

$$\begin{array}{c} Y_{it}=\alpha +\beta Treatment_{it}+\gamma Treatment_{it}\times Female_{i}+\lambda_{i}+\sum_{t=2}^{11}\omega_{t}+\epsilon_{it} \end{array}$$ (1)

where *Y*_*it*_ is firm performance as measured by real monthly profit and real monthly sales of firm *i* in wave *t*. The real profit and sales correspond to firm profit and sales that are deflated to the baseline survey time and obtained from the following questions:

1. *What was the total income the business earned DURING MONTH X after paying all expenses including wages of employees, but not including any income you paid yourself? That is, what were the PROFITS of your business DURING MONTH X.*
2. *What was the total sales DURING MONTH X of products your business makes or alters (for manufacturing firms)/of products your business did not make (for retail/trade firms)/from selling services (for service firms)?*

Following [13], the paper trims large changes in profits, in particular, the top one percent both in percentage and absolute changes. The variable of interest is *Treatment*_*it*_, which equals 1 if firm *i* received the grant in wave *t*, and equals 0 otherwise. *Female*_*i*_ equals 1 if the owner of firm *i* is female, and 0 otherwise. λ_*i*_ and *ω*_*t*_ are firm and wave fixed effects, respectively, and *ϵ*_*it*_ is the error term clustered at the enterprise level.

The coefficient *β* demonstrates the average treatment effect on the performance of male-owned firms, and *γ* shows the difference in treatment effects between male-owned firms and female-owned firms. The sum of *β* and *γ* is the overall average treatment effect on the performance of female-owned firms. This is an intention-to-treat analysis, which means that all firms assigned randomly to the treatment group are analyzed as being treated.

## Results and mechanisms

### Results

#### Effect on firm performance by gender

In this sub-section, the results of treatment impacts on firm performance by gender are provided in detail. Table 3 presents the estimated result from Eq (1). Columns (1) and (2), respectively, show the $\hat{\beta }$ and $\hat{\gamma }$ coefficients when the outcomes are real profit and real sales in the whole sample, while columns (3) and (4) indicate the corresponding estimated coefficients in the sub-sample of surviving firms. Surviving firms are firms that did not change their location and their line of business in all 11 survey waves. In general, the results from the whole sample and the sub-sample of surviving firms are quite similar for both outcome variables.

**Table 3:** Different treatment impacts on the performance of female-owned firms and male-owned firms.

	All firms		Surviving firms	
	Real profit	Real sales	Real profit	Real sales
	(1)	(2)	(3)	(4)
Treatment	1878.0***	5230.1**	1984.0***	5565.2*
	(561.1)	(2590.6)	(608.9)	(2890.0)
Treatment × Female	-1552.7**	-3782.5	-1880.5***	-4543.4
	(658.6)	(3115.0)	(670.5)	(3467.6)
Firm FE	✓	✓	✓	✓
Wave FE	✓	✓	✓	✓
Testing the overall female treatment effect = 0 (*p-values*)	0.438	0.465	0.788	0.639
*Baseline Mean Dep. Var.*	3688.3	11474.8	3659	11139.4
Observations	5427	5505	4743	4802
Number of clusters	601	602	492	493
*R* ^2^	0.032	0.017	0.034	0.020

*Note*: Standard errors, clustered at the enterprise level, are shown in parentheses. Real profits and real sales are firm profit and firm sales in LKR deflated to the baseline survey time. Firm profit is the total income that the business earned after paying all expenses including the wages of employees, but not including any income that the owners paid themselves in the month before the survey wave. Firm sales are total sales for the month before the survey wave.

*p < 0.10,

**p < 0.05,

*** *p* < 0.01.

The $\hat{\beta }$ coefficient is positive and statistically significant in all columns, which implies a positive treatment effect on the profit and sales of male-owned firms. On average, in the whole sample, male-owned firms have an increase in profit of 1,878 LKR, which is 41.5% of the baseline male-owned firm profit and 31% of the male-owned firm profit of the control group before the tsunami. Also, the treatment effect on sales is around 5,200 LKR.

With regard to gender differences in the impact of the business grant, statistically significant results are found only when real profit is the outcome variable. The sign of the $\hat{\gamma }$ coefficient is negative, which means that female-owned firms benefit less from the grant than male-owned firms. On average, their profit is around 1,553 LKR smaller than that of their male counterparts. The sum of $\hat{\beta }$ and $\hat{\gamma }$, which is the overall female treatment effect, is 326 LKR for all firms and 104 LKR for surviving firms. A statistical test is applied to the sum of the two estimated coefficients, and the result indicates that the null hypothesis of zero treatment effect on female-owned firms is not rejected in all columns. This finding is similar to [13] when they take into consideration the groups of firms in indirectly affected and unaffected zones. Given these results, real profit is the only measure of firm performance used for the remainder of the paper. Other regression results with real sales as the outcome variable are presented in Table C. in S1 File.

#### Controlling for other observable characteristics

Given that the survey was conducted four months after the tsunami and the data were collected from actively operating enterprises, there might be concerns over different characteristics between male and female entrepreneurs in the sample. Female owners and male owners might not be comparable owing to selection into self-employment and their ability to keep their businesses in operation after the tsunami. Moreover, the experimental literature emphasizes a number of key differences between women and men that might affect their economic decisions, such as risk aversion [35, 36], willingness to take risk [37], and other personality traits [38]. Therefore, a balance test on observable characteristics between female owners and male owners is administered in Table 4. Following [12], this study makes use of some of their measured characteristics of ability, personal traits, risk-taking behaviors, and locus of control.

**Table 4:** Balance test: Male owners versus female owners.

	Male		Female		
Variable	Mean	SD	Mean	SD	P-val. diff
Father’s education	7.389	(3.284)	7.433	(2.968)	(0.888)
Mother’s education	7.032	(3.063)	7.086	(3.039)	(0.863)
**Married**	0.852	(0.356)	0.771	(0.421)	**(0.010)** **
Migrant	0.116	(0.320)	0.135	(0.342)	(0.481)
Optimistic	3.083	(1.473)	3.035	(1.574)	(0.702)
Ability	-0.022	(1.188)	0.018	(1.246)	(0.695)
Experience	0.671	(0.471)	0.613	(0.488)	(0.148)
Locus of control	10.368	(1.726)	10.326	(1.759)	(0.781)
**Risk aversion**	0.344	(1.618)	-0.033	(1.463)	**(0.003)** ***
Willingness to take risk	6.564	(1.984)	6.386	(2.192)	(0.309)
Owner age	42.460	(11.232)	41.418	(11.092)	(0.250)
Financial literacy	0.576	(0.495)	0.586	(0.493)	(0.797)
**Asset index**	-0.191	(1.884)	0.233	(1.778)	**(0.004)** ***
Household size	5.026	(1.745)	4.848	(1.767)	(0.214)
Total number of workers	1.418	(0.642)	1.434	(0.623)	(0.750)
**Retail/trade**	0.463	(0.499)	0.343	(0.476)	**(0.003)** ***
Firm age	11.267	(10.867)	10.397	(10.206)	(0.312)
Interest payment	99.164	(563.624)	107.165	(585.738)	(0.864)
Asset damage	0.341	(0.475)	0.387	(0.488)	(0.235)
Observations	311		297		

*Note*: Risk aversion is measured from a lottery exercise: the higher the value, the more risk averse. Married equals 1 if the owner is married, and 0 otherwise. Asset index is the first principal component of 17 household assets. Retail/trade equals 1 if the firm belongs to the retail or trade sectors and 0 if the firm belongs to the manufacturing or service sectors.

*p < 0.10,

**p < 0.05,

***p < 0.01.

Table 4 indicates that female owners and male owners have statistically significant differences in risk aversion, civil status, firm sector and asset index. Interestingly, on average, Sri Lankan female microenterprise owners are less risk averse than their male counterparts (-0.033 versus 0.344). Those four control variables are then added to regression Eq (1) to test whether the difference in treatment effects between male-owned firms and female-owned firms is explained only by the gender of owners and not by any other observable characteristics. The regression equation is as follows:

$$\begin{array}{c} Y_{ijt}=\alpha_{1}+\beta_{1}Treatment_{ijt}+\gamma_{1}Treatment_{ijt}\times Female_{i}+\\ \sum_{j=1}^{4}\theta_{j}Treatment_{it}\times X_{ji}+\lambda_{i}+\sum_{t=2}^{11}\omega_{t}+\epsilon_{ijt} \end{array}$$ (2)

where *Y*_*ijt*_ is firm performance as measured by real monthly profit of firm *i* in wave *t*, of which the owner has characteristics *j*. *Treatment*_*ijt*_ equals 1 if firm *i*, of which the owner has characteristics *j*, received the grant in wave *t*. *Female*_*i*_ equals 1 if the owner of firm *i* is female and 0 otherwise. *X*_*ji*_ refers to characteristics *j* of the owner of firm *i* or firm *i* (j = 1,2,3,4), which include Risk aversion, Married, Asset index and Retail/trade. *Risk aversion* is measured from a lottery game played with real money by each entrepreneur in the second wave (see [12] for more details). *Married* is a dummy variable that equals 1 if the owner is married, and 0 otherwise. *Asset index* is the first principal component of 17 household assets. *Retail/trade* is a dummy variable that equals 1 if the firm belongs to the retail or trade sectors and 0 if the firm belongs to the manufacturing or service sectors. λ_*i*_ and *ω*_*t*_ are firm and wave fixed effects, and *ϵ*_*ijt*_ is the error term clustered at the enterprise level.

Table 5 presents the estimated results of Eq (2). Columns (2), (3), (4) and (5) contain the interaction term between the treatment variable and each control variable, while Column (6) contains all of the interaction terms included in the regression. The results are very consistent with the baseline results, which indicates a statistically significant and positive treatment effect on the profits of male-owned firms, and zero effect on those of female-owned firms. Moreover, the coefficients of the interaction terms between the treatment and all four control variables are insignificant, which suggests that the different treatment effects between male-owned firms and female-owned firms are explained by the gender of owners and not by other observable characteristics.

**Table 5:** Difference in treatment effects after controlling for other observable characteristics.

	Dependent variable is real profit					
	(1)	(2)	(3)	(4)	(5)	(6)
Treatment	1878.0***	1897.0***	1550.4*	1912.6***	1947.0***	1639.1*
	(561.1)	(589.0)	(837.8)	(561.6)	(591.5)	(908.6)
Treatment×Female	-1552.7**	-1569.7**	-1514.9**	-1548.3**	-1571.1**	-1543.9**
	(658.6)	(673.0)	(628.9)	(657.4)	(650.3)	(638.5)
Treatment × Risk aversion		-41.13				-50.53
		(238.7)				(241.5)
Treatment × Married			375.6			407.1
			(851.3)			(867.3)
Treatment×Asset Index				-138.6		-138.0
				(258.1)		(258.2)
Treatment×Retail/Trade					-124.3	-105.4
					(545.6)	(535.7)
Firm FE	✓	✓	✓	✓	✓	✓
Wave FE	✓	✓	✓	✓	✓	✓
Testing the overall female treatment effect = 0 *(p-values)*	0.438	0.437	0.969	0.394	0.437	0.921
*Baseline Mean Dep. Var.*	3688.3	3688.3	3688.3	3688.3	3688.3	3688.3
Observations	5427	5427	5427	5427	5426	5426
Number of clusters	601	601	601	601	601	601
*R* ^2^	0.032	0.032	0.032	0.032	0.032	0.032

*Note*: Standard errors, clustered at the enterprise level, are shown in parentheses. Real profit is the dependent variable and defined as it is in Table 3. Risk aversion is measured from a lottery game played with real money by each entrepreneur in wave 2 (see [12] for more details). Married is a dummy variable that equals 1 if the owner is married, and 0 otherwise. Asset index is the first principal component of 17 household assets. Retail/trade is a dummy variable that equals 1 if the firm belongs to the retail or trade sectors and 0 if the firm belongs to the manufacturing or service sectors.

*p < 0.10,

**p < 0.05,

***p < 0.01.

#### Heterogeneity in treatment effects

In this sub-section, the study examines whether the baseline result is heterogeneous across different subgroups of firms in the experiment. The heterogeneity of the treatment effect is investigated first through the two treatment amounts. Table B. in S1 File suggests that there is no statistically significant difference in the average treatment effects between the two methods from the same treatment amount. Hence, the two treatment methods (cash and in-kind) are pooled in each treatment amount. The study uses two new binary treatment variables, which are *Treatment 10000* and *Treatment 20000* to compare the average treatment effects between the subgroup receiving 10,000 LKR and the subgroup receiving 20,000 LKR, respectively, with the control group.

The balance tests on observable characteristics between each subgroup and the control group are conducted to verify randomization by gender in Tables D and E in S1 File. The results suggest that the two treatment subgroups are quite comparable to the control group despite a significant decrease in the number of observations. Regression Eq (1) with the two new variables of interest is then used to estimate the average treatment effects. Table 6 shows the estimated result with the two new variables of interest.

**Table 6:** Heterogeneity in treatment effects.

	Dependent variable is real profit			
	(1)	(2)	(3)	(4)
Treatment 10000	2296.0***			
	(780.0)			
Treatment 10000 × Female	-1710.4**			
	(859.1)			
Treatment 20000		1438.2*		
		(744.6)		
Treatment 20000 × Female		-1750.5*		
		(1027.7)		
Treatment			2191.3***	1435.2***
			(651.3)	(514.2)
Treatment × Female			-2048.4**	-1708.2***
			(797.8)	(587.2)
Treatment × Retail/trade			-571.0	
			(834.8)	
Treatment × Retail/trade × Female			1015.5	
			(1077.1)	
Treatment × Asset damage				1835.3
				(1632.2)
Treatment × Asset damage × Female				460.3
				(1933.8)
Firm FE	✓	✓	✓	✓
Wave FE	✓	✓	✓	✓
Testing the overall female treatment effect: = 0 *(p-values)*	0.193	0.684	0.783	0.461
			*(Manu/Serv)*	*(W/o damage)*
			0.288	0.001
			*(Retail/Trade)*	*(W/ damage)*
Observations	4280	3462	5426	5427
Number of clusters	479	386	601	601
*R* ^2^	0.030	0.028	0.032	0.033

*Note*: Standard errors, clustered at the enterprise level, are shown in parentheses. Real profit is the dependent variable and defined as it is in Table 3. Treatment 10000 is a dummy variable that equals 1 if a firm received 10,000 LKR in cash or in-kind in wave *t*, and 0 if a firm did not receive either 10,000 LKR or 20,000 LKR in wave *t*. Treatment 20000 is a dummy variable that equals 1 if a firm received 20,000 LKR in cash or in-kind in wave *t*, and 0 if a firm did not receive either 10,000 LKR or 20,000 LKR in wave *t*. Retail/trade equals 1 if a firm belongs to the retail or trade sectors and 0 if a firm belongs to the manufacturing or service sectors. Asset damage equals 1 if a firm had asset damage caused by the tsunami, and 0 otherwise.

*p < 0.10,

**p < 0.05,

***p < 0.01.

Columns (1) and (2) of Table 6 show similar results to Table 3, in which the business grant has a positive effect on the profits of male-owned firms, and zero effect on their female counterparts for both treatment amounts. Interestingly, the higher treatment amount (20,000 LKR) has a lower effect on male-owned firms than the smaller treatment amount (10,000 LKR) (2,296 versus 1,438.2). This finding is quite counter-intuitive when the 20,000 LKR treatment yields an impact equivalent to only 63% of the impact from the 10,000 LKR treatment. With regard to gender differences, the coefficients of interaction terms between the two new treatment variables and *Female* are negative, significant and very similar in magnitude (-1,710.4 and -1,750.5).

In columns (3) and (4), the differences in average treatment effects are then examined across sectors and levels of business asset damage. Column (3) focuses on the heterogeneity of the treatment effects and gender disparity by sector. In particular, the results for firms in manufacturing and service sectors are in line with the baseline results (positive for male-owned firms: 2,191.3; and negative for the gender gap: -2,048.4). In regard to firms in the retail or trade sectors, the positive coefficient of *Treatment* × *Retail*/*Trade* × *Female* (1,015.5) implies that female-owned firms benefit more than male-owned firms from receiving the business grant; however, it is not a significant result.

Column (4) reports the same pattern as the baseline results in the subgroup of firms without business asset damage (1,435.2 for males and -1,708.2 for gender difference). However, there is no significant result in the subgroup of firms with asset damage. The overall effect on females is tested in both subgroups, and the result implies that the null hypothesis of zero effect on the profits of female-owned firms in the subgroup without asset damage is not rejected (p-value = 0.461). However, the overall effect on females in the subgroup with asset damage is different from zero. Given this result, the mean treatment effects on male and female-owned firms from the subgroup of firms with asset damage are then estimated separately in Table F. in S1 File. The results indicate that in this subgroup, both male and female-owned firms benefit from the business grant. However, in terms of magnitude, male-owned firms benefit more from the grant than female-owned firms (2,947 LKR versus 2,142.4 LKR, respectively). This finding implies that when firms experience direct economic losses, both male and female owners appear to use the business grant effectively to rebuild their businesses.

### Mechanisms

In this section, the paper explores potential mechanisms behind the main results. Table 6 shows that in terms of magnitude, the treatment effects on male-owned firms and the gender differences in treatment effects are quite different between the two subgroups receiving 10,000 LKR and 20,000 LKR. Moreover, the effects are different from the baseline results. In particular, the treatment effects on the performance of male-owned enterprises are 1,878 LKR for all firms, 2,296 LKR for the subgroup receiving 10,000 LKR, and 1,438.2 LKR for the subgroup receiving 20,000 LKR, while the corresponding gender differences are 1,552.7, 1,710.4 and 1,750.5 LKR, respectively. These results suggest proceeding with a separate analysis for each treatment amount.

#### Gender differences in business investment and household expenditure

First, the paper focuses on the business investment behaviors of microentrepreneurs following the tsunami. The outcome variables are the monthly investment in capital stock without land, input purchases and interest paid on loans. The variables of interest include *Treatment 10000* and *Treatment 20000*. Importantly, sectors might play a role in business investment behaviors. Therefore, the interaction term between *Retail/trade* and wave fixed effects is added to allow for the different time paths of capital stock, input purchases and interest payments of firms in different sectors.

Table 7 provides evidence on gender differences in business investment. Columns (1) and (3) suggest that in the subgroup receiving 10,000 LKR, the business grant has a significant and positive impact on the investment of male-owned firms in capital stock and inputs (28,699.5 and 5,259.6 LKR, respectively). Moreover, female owners invest less in their businesses than male owners (-23,290 for capital stock and -5,083.8 for input purchases).

**Table 7:** Gender differences in business investment.

	Capital stock		Input purchases		Interest payment	
	(1)	(2)	(3)	(4)	(5)	(6)
Treatment 10000	28699.5***		5259.6*		11.41	
	(10371.2)		(2937.7)		(32.39)	
Treatment 10000 × Female	-23290.0**		-5083.8*		113.5*	
	(11506.1)		(2908.6)		(68.05)	
Treatment 20000		10457.5***		-1330.2		-15.20
		(3585.4)		(3058.5)		(20.36)
Treatment 20000 × Female		14341.8		6148.2		-20.50
		(10824.1)		(5055.3)		(28.63)
Firm FE	✓	✓	✓	✓	✓	✓
Wave FE × Retail/trade	✓	✓	✓	✓	✓	✓
Observations	3989	3229	4262	3447	4133	3343
Number of clusters	472	379	479	386	477	384
*R* ^2^	0.091	0.109	0.021	0.028	0.017	0.015

*Note*: Standard errors, clustered at the owner level, are shown in parentheses. Capital stock is monthly firm capital stock without land (including equipment and inventories minus equipment rent). Input purchases are monthly raw material expenditure for manufacturing firms and items for resale for retail or trade and service firms. Interest payment is monthly interest paid on loans, which is a category of business expenses. Treatment 10000 and Treatment 20000 are defined as they are in Table 6.

*p < 0.10,

**p < 0.05,

***p < 0.01.

Columns (2) and (4) show the estimated results for the subgroup receiving the higher treatment amount. Column (2) indicates that the treatment effect on male owners’ investments in capital stock is significant and positive. However, they invest less than male owners in the subgroup receiving 10,000 LKR (10,457.5 versus 28,699.5 LKR, or approximately one-third). This finding is consistent with the result in Table 5 that the higher treatment amount has a lower effect than the smaller treatment amount on the profits of male-owned firms. In addition, Table G. in S1 File suggests that male owners in the subgroup receiving the lower treatment amount spend more time working (6.785 hours per week). However, there is no significant treatment impact in weekly working hours of male owners in the subgroup receiving the higher treatment amount. Therefore, the interesting result of the smaller treatment effect resulting from the higher treatment amount might be explained by the difference in investing grants in capital stock and in work effort between male owners in the two subgroups.

With respect to gender differences in the subgroup receiving 20,000 LKR, the estimated coefficients of the interaction term between *Treatment 20000* and *Female* in columns (2) and (4) are positive, which suggests that female owners invest more in capital stock and purchase more inputs than male owners, but the estimates are not statistically significant. This finding implies that even though female owners invest more in their businesses, their investments cannot be translated into higher profits or better performance. Columns (5) and (6) report the findings on gender differences in monthly interest paid on loans. The results indicate that female owners receiving 10,000 LKR repay loans more than their male counterparts (113.5 LKR per month). However, there is no evidence of gender differences in the subgroup receiving the higher treatment amount.

Next, the study examines whether there is any difference in household expenditure between male and female owners by using data from a complementary household survey. In light of the context following the tsunami, their expenditure on household basic needs is defined as monthly expenditure on food consumption, housing, healthcare and clothing. Moreover, the related literature supports the notion that when women increase their share of household income, they spend more on their children [39, 40]. Therefore, the paper also focuses on monthly expenditure on education, which consists of school supplies, school fees and donations.

Table 8 provides the estimated results of gender differences in household expenditure. Columns (1) and (2) present findings when expenditure on basic needs is the dependent variable. Column (1) indicates that there are no significant gender differences in the subgroup receiving the smaller treatment amount. In Column (2), the positive and significant coefficient (1,854.3 LKR) of the interaction term between *Treatment 20000* and *Female* implies that female owners in the subgroup receiving the higher treatment amount spend more than male owners do on their household basic needs. In columns (3) and (4), when expenditure on education is the outcome variable, there are no significant results in either the treatment effects on male owners or the gender differences in the treatment effects. This finding is similar to [13], and the lack of significant results on educational spending might arise from the inexpensive system of schooling in Sri Lanka.

**Table 8:** Gender differences in household expenditure.

	Basic needs		Education	
	(1)	(2)	(3)	(4)
Treatment 10000	-154.2		-150.5	
	(893.2)		(105.7)	
Treatment 10000 × Female	-180.0		55.68	
	(871.3)		(113.2)	
Treatment 20000		193.6		29.57
		(949.7)		(80.23)
Treatment 20000 × Female		1854.3*		-84.39
		(1090.4)		(108.5)
Individual FE	✓	✓	✓	✓
Wave FE	✓	✓	✓	✓
Observations	1690	1361	1690	1361
Number of clusters	460	370	460	370
*R* ^2^	0.001	0.013	0.007	0.009

*Note*: Standard errors, clustered at the owner level, are shown in parentheses. Basic needs is a monthly expenditure that includes food consumption (the expenditure on groceries, food consumed at home and food consumed outside the home), housing (house rent, taxes, maintenance, water bill), healthcare and clothing. Education is a monthly expenditure that includes school supplies, school fees and donations.

*p < 0.10,

**p < 0.05,

***p < 0.01.

Overall, the finding that female-owned firms benefit less from the grant than their male counterparts can be explained by the different behaviors of male owners and female owners in business investment and household expenditure. More specifically, female owners in the subgroup receiving 10,000 LKR invest less in their businesses and pay more interest on firm loans. In the subgroup receiving 20,000 LKR, female owners invest more in their businesses and spend more on their household basic needs. However, their higher business investments cannot be translated into higher profit.

#### Gender differences in initial business closures

Following natural disasters, firms experience several barriers such as supply chain disruptions [34], loss of equipment, and loss of staff and customers. These adverse impacts deteriorate business activities and even force businesses to close. In this sub-section, the paper investigates whether there are any gender differences in initial business closures that can potentially explain the difference in treatment effects on the performance of female and male-owned firms.

First, the question *“Are you working in the same line of business and in the same location as you were working in when we interviewed you 3 months ago?”* is used across survey waves to identify whether an owner has closed their initial business. In the sample, it is possible to identify the status of 590 firms (out of 608 firms), including 290 female-owned firms (161 treatment and 129 control units) and 300 male-owned firms (172 treatment and 128 control units). Out of all female owners, 35 treated and 15 control owners shut down their initial businesses, which account for 21.6% and 11.1% of the female treatment and control groups, respectively. In the male sub-sample, 23 treated and 23 control owners closed their baseline firms. These represent 13.1% and 17% of the corresponding male treatment and control groups.

The following regression equation is then estimated:

$$\begin{array}{c} Close_{i}=\phi_{0}+\phi_{1}D_{i}+\phi_{2}Female_{i}+\phi_{3}D_{i}\times Female_{i}+\delta X_{i}+\xi_{i} \end{array}$$ (3)

where *Close*_*i*_ is a dummy variable that equals 1 if the owner of firm *i* changed their line of business or changed both their line of business and their location or was no longer self employed or was not engaged in business activity, and 0 otherwise. *D*_*i*_ is the variable of interest, either *Ever Treatment 10000* or *Ever Treatment 20000* that equals 1 if firm *i* received 10,000 LKR or 20,000 LKR, and 0 when firm *i* did not receive either 10,000 LKR or 20,000 LKR from the experiment. *Female*_*i*_ indicates the gender of the owner of firm *i*, which equals 1 if the owner is female and 0 if the owner is male. *X*_*i*_ consists of control variables that represent the owner characteristics and the firm characteristics of firm *i* (risk aversion, civil status, asset index and firm sector).

Next, a survival analysis is conducted to estimate the lifespan of baseline microenterprises. The event of interest is the closure of initial business. Time of origin is the time of the baseline survey, and time to event is the number of months between the time of event and the time of origin. The question *“When did you stop working in this business? Day, month”* is used to identify the *Time to event*. The study then employs a parametric regression survival time model, in which the *Time to event* is assumed to be a function of explanatory variables. The specification is as follows:

$$\begin{array}{c} log\left(Time_{i}\right)=\rho_{0}+\rho_{1}D_{i}+\rho_{2}Female_{i}+\rho_{3}D_{i}\times Female_{i}+\pi X_{i}+\iota_{i} \end{array}$$ (4)

where *Time*_*i*_ is the number of months between the baseline time and the time of event when firm *i* was shut down. All independent variables in Eq (4) are defined as they are in Eq (3), and *ι*_*i*_ is the error term.

Table 9 presents the regression results of equations (3) and (4) when the two treatment amounts are analyzed separately. The estimation of Eq (3) by logistic regression model and linear probability model is reported from columns (1) to (4). The positive and significant $\hat{\phi_{3}}$ coefficients in columns (1) and (3) (1.293 and 0.176) indicate that in the subgroup receiving 10,000 LKR, female owners are more likely to close their initial businesses, whereas there is no significant evidence for the subgroup receiving 20,000 LKR in columns (2) and (4). More specifically, the result in column (3) implies that female recipients in the subgroup receiving the lower amount have a 17.6 percentage point increase in the likelihood of closing their initial businesses. This finding provides a potential explanation for why they invest less in their businesses than male owners in Table 7.

**Table 9:** Gender differences in initial business closures.

	Logit		Linear probability model		Survival analysis	
	Close	Close	Close	Close	Time	Time
	(1)	(2)	(3)	(4)	(5)	(6)
Female	-0.443	-0.431	-0.054	-0.052	-0.460	-0.437
	(0.362)	(0.366)	(0.044)	(0.044)	(0.344)	(0.347)
Ever Treatment 10000	-0.446		-0.059		-0.501	
	(0.391)		(0.05)		(0.363)	
Ever Treatment 10000 × Female	1.293**		0.176**		1.254***	
	(0.526)		(0.069)		(0.481)	
Ever Treatment 20000		-0.438		-0.055		-0.569
		(0.424)		(0.053)		(0.407)
Ever Treatment 20000 × Female		0.819		0.101		0.847
		(0.643)		(0.081)		(0.589)
Controls	✓	✓	✓	✓	✓	✓
Observations	470	377	470	377	470	376

*Note*: Robust standard errors are shown in parentheses. Close is a dummy variable that equals 1 if the owner changed their line of business/changed both their line of business and their location/was no longer self employed/was not engaged in business activity, and 0 otherwise. Time is the number of months between the baseline time and the time when the owner closed their initial business. Ever Treatment 10000 is the binary treatment variable that equals 1 if the firm ever received 10,000 LKR in cash or business equipment and equals 0 if the firm did not receive either 10,000 LKR or 20,000 LKR from the experiment. Ever Treatment 20000 is the binary treatment variable that equals 1 if the firm ever received 20,000 LKR in cash or business equipment and equals 0 if the firm did not receive either 10,000 LKR or 20,000 LKR from the experiment. Control variables include Married, Asset index, Retail/trade, Risk aversion.

*p < 0.10,

**p < 0.05,

***p < 0.01.

Columns (5) and (6) display the result of the survival analysis on the assumption that the hazard of an event is constant over time. The estimate (1.254) indicates that in the subgroup receiving 10,000 LKR, female-owned firms have a higher hazard rate or shorter survival time than male-owned firms. This is a strong assumption; hence it is important to test the regression equation with another survival distribution. Table H. in S1 File provides a robustness check when the study applies the Weibull distribution, which allows the hazard rate to change over time, and the results still hold. The estimated effects when the two treatment amounts are pooled are reported in Table I. in S1 File.

#### Gender differences in psychological recovery

In this sub-section, the study explores whether the mental well-being of business owners following the tsunami can be a mechanism that drives the results relating to gender differences in the treatment impact on firm performance. The related literature points to a positive relationship between receiving grants and the mental health of recipients, including [23, 27, 41]. Moreover, there is some evidence of a link between entrepreneurs’ mental health and firm performance. Wincent et al. [42] and Hessels et al. [43] show that business owners with good mental health and well-being are more likely to endure and have better firm performance. In addition, Parida [44] reports that in the context of post-disaster recovery, women are more likely than men to suffer from mental health issues. This evidence suggests that gender differences in the impact of receiving grants on the psychological recovery of business owners may provide an explanation for the main results.

The following Likert scale questions are used to measure the psychological status of respondents from the first to the ninth survey wave:

*For each of the following, say whether you strongly agree, agree, disagree or strongly disagree with the following statements as applied to your life:*

1. *I no longer talk about the tsunami these days (1 = strongly agree; 2 = agree; 3 = disagree; 4 = strongly disagree)*
2. *I have changed my outlook on life as a result of the tsunami (1 = strongly disagree; 2 = disagree; 3 = agree, 4 = strongly agree)*

The first question reveals how people retreat from natural disasters, while the second question reflects their struggle to accept the event [45]. A smaller value in both Likert scales indicates better psychological status. Since outcomes are ordered categorical variables, the study employs a random-effect generalized ordered probit model with an auto-fit procedure developed by Pfarr et al. [46] to examine treatment effects.

In the traditional ordered probit model, all estimated coefficients are assumed not to vary between categories, i.e the parallel-lines assumption. This is a very strong assumption and frequently violated in practice [47]. The advantage of a random-effect generalized ordered probit model is that it provides a more flexible approach than the traditional model that allows for heterogeneous effects of explanatory variables.

*Regression framework*

Let y* be a latent variable, which is observed in discrete form through a censoring rule:

$$\begin{array}{c} y=\{\begin{array}{c} 1\;\text{if}\;\mu_{0}<y^{*}\leq \mu_{1}\\ 2\;\text{if}\;\mu_{1}<y^{*}\leq \mu_{2}\\ 3\;\text{if}\;\mu_{2}<y^{*}\leq \mu_{3}\\ 4\;\text{if}\;\mu_{3}<y^{*}\leq \mu_{4} \end{array}\; \end{array}$$ (5)

where y is the response to question (1) or (2) that has the value of 1, 2, 3 or 4. *μ*_*j*_ are unknown threshold parameters (j = 1,2,3,4). y* is defined as the function of a set of covariates *Z* and the error term *υ*, which is assumed to be normally distributed:

$$\begin{array}{c} y^{*}=Z^{\prime }\kappa +\upsilon \end{array}$$ (6)

*Z* includes the variable of interest *T_*it*_*, which can be one variable from the set (*Treatment_*it*_*, *Treatment 10000_*it*_*, *Treatment 20000_*it*_*); the interaction term between the variable of interest and *Female*_*i*_; and other control variables (dummy variables for dead relatives, injured household members as a result of the tsunami and whether the owner was hit by water during the tsunami). Moreover, *μ*_*j*_ is allowed to depend on the covariates:

$$\begin{array}{c} \mu_{j}=\tilde{\mu_{j}}+Z^{\prime }\tau_{j} \end{array}$$ (7)

*τ*_*j*_ represents the impact of the covariates on the thresholds.

The cumulative probability of the generalized ordered probit model is expressed as follows:

$$\begin{array}{c} Pr\left(y\leq j|Z\right)=F(\tilde{\mu_{j}}-Z^{\prime }\left(\kappa -\tau_{j}\right)) \end{array}$$ (8)

where F is a cumulative standard normal distribution, j = 1, 2, 3, 4. Let *κ*_*j*_ = *κ* − *τ*_*j*_, then the model has a specific *Z*′*κ*_*j*_ for each category *j* of the outcome variables.

For panel data, the random-effect generalized ordered probit model takes into consideration the individual effects λ_*i*_. The individual effects are assumed to have zero means and a constant variance. It requires that *κ*_*j*_ > *κ*_*j*−1_, hence *κ*_0_ = −∞, *κ*_4_ = ∞, or *F*(−∞) = 0, *F*(∞) = 1. Therefore, the outcome probabilities are as follows:

$$\begin{array}{c} Pr\left(y_{it}=1|Z_{it},\lambda_{i}\right)=F\left(-Z_{it}^{\prime }\kappa_{1}-\lambda_{i}\right) \end{array}$$

$$\begin{array}{c} Pr\left(y_{it}=j|Z_{it},\lambda_{i}\right)=F\left(-Z_{it}^{\prime }\kappa_{j}-\lambda_{i}\right)-F\left(-Z_{it}^{\prime }\kappa_{j-1}-\lambda_{i}\right)\;\text{for}\;\text{j}=2,3 \end{array}$$

$$\begin{array}{c} Pr\left(y_{it}=4|Z_{it},\lambda_{i}\right)=1-F\left(-Z_{it}^{\prime }\kappa_{3}-\lambda_{i}\right) \end{array}$$

This process leads to the estimation of three binary probit models, including category 1 versus categories 2–4, categories 1–2 versus categories 3–4 and categories 1–3 versus category 4.

*Regression result*

Table 10 presents the regression results of the random-effect generalized ordered probit model with an auto-fit procedure. In this analysis, the study focuses on the binary probit model that compares the choice between categories 1–2 versus 3–4 (strongly agree and agree versus strongly disagree and disagree for question [1] and the opposite direction for question [2]). The coefficients of *Treatment* are strongly significant and negative in columns (1) and (5) (-0.162 and -0.129), which implies that the owners in the treatment group are more likely to report better psychological status. For the interaction term between *Treatment* and *Female*, both columns (2) and (6) report positive coefficients (0.092 and 0.101), which indicates that female owners are more likely to report worse mental status. However, these results are not statistically significant.

**Table 10:** Impacts of treatment on psychological recovery.

	No longer talk about tsunami				Change outlook due to tsunami			
	(1)	(2)	(3)	(4)	(5)	(6)	(7)	(8)
*Category 1–2 vs 3–4*								
Treatment	-0.162***	-0.205***			-0.129**	-0.174**		
	(0.061)	(0.074)			(0.065)	(0.077)		
Treatment × Female		0.092				0.101		
		(0.092)				(0.094)		
Treatment 10000			-0.227**				-0.230**	
			(0.095)				(0.097)	
Treatment 10000 × Female			0.037				0.125	
			(0.117)				(0.117)	
Treatment 20000				-0.111				0.012
				(0.096)				(0.102)
Treatment 20000 × Female				0.212				-0.050
				(0.154)				(0.167)
Relatives dead	0.114	0.104	0.133	-0.009	-0.078	-0.087	-0.104	-0.009
	(0.086)	(0.086)	(0.097)	(0.107)	(0.090)	(0.091)	(0.105)	(0.111)
Hit by tsunami	0.150*	0.154*	0.136	0.235**	0.472***	0.478***	0.472***	0.413***
	(0.080)	(0.080)	(0.092)	(0.095)	(0.090)	(0.090)	(0.103)	(0.110)
Household member	0.146	0.144	0.063	0.116	0.365***	0.362***	0.475***	0.316**
injured	(0.107)	(0.106)	(0.129)	(0.131)	(0.125)	(0.126)	(0.150)	(0.140)
Wald Test	0.274	0.333	0.196	0.286	0.430	0.518	0.168	0.111
Observations	4510	4510	3556	2882	4510	4510	3556	2882

*Note*: Standard errors are shown in parentheses. Treatment 10000 and Treatment 20000 are defined as they are in Table 6. Hit by tsunami equals 1 if the owner was hit by water during the tsunami, and 0 otherwise. Relatives dead equals 1 if the owner had at least one relative killed in the tsunami, and 0 otherwise. Household member injured equals 1 if at least one member of the owner’s household was injured because of the tsunami, and 0 otherwise.

*p < 0.10,

**p < 0.05,

***p < 0.01.

When each treatment amount is analyzed separately, the coefficients of *Treatment 10000* in columns (3) and (7) are negative and significant, which implies that the male owners in the subgroup receiving 10,000 LKR have a higher likelihood of reporting better mental recovery. However, the results for male owners in the subgroup receiving 20,000 LKR are inconclusive, with a negative coefficient (-0.111) in column (4) and a positive coefficient (0.012) in column (8); neither is significant. In addition, all estimated coefficients of the interaction terms between *Treatment*, *Treatment 10000* or *Treatment 20000* and *Female* are not statistically significant, hence there is no evidence supporting gender differences in the treatment impact on psychological recovery. Table 10 also includes the Wald tests of the parallel lines assumption. The test statistic in all columns is insignificant at the level of 0.05, which assures that the parallel lines assumption is not violated. Hence, the results are credible.

### External validity

The main results of the paper suggest that there are gender differences in the treatment effect when female-owned firms and male-owned firms receive a similar business grant following natural disasters. However, this finding is based on the context of a single country. One question might arise as to the generalizability of the results, namely this: does the causal effect hold in other settings, treatments and outcomes? COVID-19 provides a context to check the external validity of the main results because it is considered to be a natural hazard according to the classification of the International Federation of Red Cross and Red Crescent Societies (IFRC).

In this sub-section, the study tests for external validity by using firm-level data from the World Bank Enterprise Surveys and COVID-19 follow-up surveys. According to the definition of the World Bank Group, microenterprises are enterprises with between 0 and 10 employees. Therefore, the sample is restricted to firms from developing countries that have less than 10 employees and receive business supports (cash or non-cash). The final sample includes 377 microenterprises from 22 developing countries in three sectors: manufacturing, retail and services. The list of 22 developing countries consists of Armenia, Azerbaijan, Belarus, Bosnia & Herzegovina, Chad, Croatia, Cyprus, El Savador, Georgia, Guatemala, Guinea, Hungary, Jordan, Kazakhstan, North Macedonia, Malta, Moldova, Montenegro, Morocco, Romania, Serbia and Zimbabwe.

The outcome variable is the change in sales in the last month prior to the survey compared to the same month of the previous year, and it is measured in percentages. The change in sales receives a positive value if sales increase, 0 if sales remain the same and a negative value if sales decrease. Note that the COVID follow up surveys were implemented over different periods in different countries. Out of the 22 developing countries, the World Bank Group carried out the COVID follow up surveys in 16 of them in 2020. Therefore, the reference month of the previous year is a month in 2019, which was prior to COVID-19. In the other six countries, the survey was administered in 2021, and the reference months are mainly January, February and March 2020 before the World Health Organization (WHO) declared that COVID-19 was a pandemic.

The cross-sectional regression equation is as follows:

$$\begin{array}{c} \Delta sales_{isj}=\kappa +\delta Female_{i}+\gamma X_{i}+\eta_{s}+\mu_{j}+\upsilon_{isj} \end{array}$$ (9)

where *Δsales*_*isj*_ is the monthly change in the sales of firm *i* in sector *s* in country *j*, *Female*_*i*_ is a dummy variable, which equals 1 if the main owner of firm *i* is female and 0 otherwise, *X*_*i*_ are control variables, *η*_*s*_ are sector fixed effects, *μ*_*j*_ are country fixed effects, and *υ*_*isj*_ is the error term. The sector and country dummies are included in order to control for differences across sectors and countries.

Table 11 presents the estimated results of Eq (9). In column (1), the $\hat{\delta }$ coefficient (-9.606) is negative and statistically significant, which implies that the sales of female-owned microenterprises decrease more than those of male-owned microenterprises. The results remain stable in terms of sign and significance when more control variables (firm age, labor and firm working hours) are added from column (2) to column (4). On average, the sales of female-owned firms decrease 8.73% more than those of their male counterparts, which represents 21.3% over the sample mean. Since data on firm profits after the appearance of COVID-19 are not available, it is impossible to examine the same outcome in this period. However, the findings in this sub-section provide suggestive evidence that gender differences in treatment effects on firm performance following natural disasters appear to hold in other contexts, and are not limited to Sri Lanka.

**Table 11:** External validity of the main results in the context of the COVID-19 pandemic.

	Dependent variable is the monthly change in sales			
	(1)	(2)	(3)	(4)
Female	-9.606***	-9.591***	-8.806**	-6.936**
	(3.675)	(3.682)	(3.694)	(3.394)
Firm age		0.236	0.230	0.319**
		(0.148)	(0.142)	(0.133)
Labor			3.149***	2.422***
			(0.801)	(0.694)
Working hours				-23.80***
				(3.266)
Country FE	✓	✓	✓	✓
Sector FE	✓	✓	✓	✓
*Mean Dep. Var.*	-40.95	-40.95	-40.95	-40.95
Observations	377	376	376	376
*R* ^2^	0.365	0.371	0.399	0.494

*Note*: Robust standard errors are shown in parentheses. Dependent variable is the change in sales in the last month prior to the survey compared to the same month of the previous year, measured in percentages. Firm age is the difference between 2021 and the year that the firm started operations. Labor is the number of permanent full time employees in the last month before the survey. Working hours are the establishment’s total hours worked per week in the last month before the survey compared to the same month in 2019 (1 = increase, 2 = remain the same, 3 = decrease). Data are collected from 22 developing countries in three sectors: manufacturing, retail and services.

*p < 0.10,

**p < 0.05,

***p < 0.01.

## Discussion and conclusion

Natural disasters are sharply increasing in both frequency and severity around the world. According to the latest report from the Geneva-based IFRC, more than 100 natural disasters have occurred since March 2020 when the WHO declared that the COVID-19 outbreak was a global pandemic [48]. There has been some good news regarding the vaccine for COVID-19; however, as the IFRC Secretary General Jagan Chapagain mentioned, *“Unfortunately, there is no vaccine for climate change”*. Therefore, the post-disaster recovery of vulnerable communities, especially small businesses, should receive special attention from both governmental and non-governmental organizations.

This study provides evidence relating to the different effects of a business grant on the performance of male-owned firms and female-owned firms in Sri Lanka following the 2004 Indian Ocean tsunami. The results highlight that only male-owned firms benefit from the business grant, while the treatment effects on the profits of female-owned firms is zero. Several studies in the experimental literature have reported gender differences in treatment effects on firm performance [17–19], but only under normal circumstances. This paper contributes to the literature by providing new evidence in the context of post-disasters. The main findings are consistent with the results of previous empirical studies, but not in line with the predictions of liberal feminist theory and social feminist theory.

Even though the main results differ from the prediction of social feminist theory, the present study provides some potential mechanisms that are related to expectations from the theory. More specifically, social feminist theory argues that there are differences in traits and experiences between men and women, which lead to different behaviors that might affect their firm performance. The paper documents gender differences in business investment, interest payment, household expenditure and the likelihood of closing their initial businesses. In addition, the paper shows a positive treatment effect on the psychological recovery of microentrepreneurs. This finding, which is the first experimental evidence relating to small business owners who receive grants, is in line with several previous studies that document a positive relationship between cash transfers and mental health outcomes for children, adolescents and households in developing countries [23–28].

This study has a number of limitations, in particular regarding the data. First, the data pertain only to microenterprises actively operating in May 2005, which was four months after the tsunami. There is a possibility that female-owned enterprises and male-owned enterprises shut down their businesses at different rates before the baseline survey. Moreover, female owners and male owners might have different processes to select themselves into self-employment. Therefore, there might be concerns over different characteristics between male and female owners in the sample. Despite conducting a balance test on the observable characteristics of female and male owners (see Table 4), this might not completely resolve the issue.

Second, the follow-up survey in 2010 suggests that out of the 25 female owners that closed their initial businesses in the subgroup receiving 10,000 LKR, 52% reopened their initial businesses (8) or operated different businesses (5), 16% switched to be employees (4) and 32% chose to do housework or take care of their families (8). The main reason that 16% switched to work for a wage was to have a more stable working environment with less stress and better working hours. For the 32% that chose to do housework or take care of their families, they made the decision because of their business losses, their health problems, and the need to take care of children and their family members. For the five female owners that operated different businesses, their main reasons were a lack of money to open in their favorite sectors and the flexibility that the new sector offered them to look after family members. However, there is no information about the reason why the other eight female owners shut down and then reopened their businesses. This is an area where the survey is not able to capture some important changes. Therefore, the addition of qualitative data from interviews or focus groups might help to elucidate changes in social and gender norms that could not be captured by the quantitative survey, and provide contextual data on how and why those changes occur.

Lastly, the paper provides suggestive evidence on the external validity of the main results by using firm-level data from the COVID-19 period. However, it is impossible to check the external validity using the same outcome (firm profit) since there are no available data. As additional data from the World Bank Enterprise Survey become available in the near future, it will be possible to examine whether the main results hold with firm performance as measured by profit in the context of COVID-19.

The main results have implications for business recovery programs aimed at supporting female microentrepreneurs following natural disasters. When both male and female-owned firms receive a similar business grant, on average, only the male-owned firms improve their performance, while the treatment impact on the performance of the female-owned firms is zero. In addition, the treatment amount has been shown to play an important role in how female small business owners behave and make decisions. The potential reasons for the main results are that (i) female owners invest less in their businesses in the subgroup receiving the smaller amount and spend more on their households in the subgroup receiving the higher amount, and (ii) female owners are more likely to close their initial businesses in the subgroup receiving the lower amount. If the first reason is due to women’s preferences and behaviors, then it is difficult to undertake any intervention that changes the situation. With regard to the second reason, it might be solved by interventions that help female-owned firms to increase their likelihood of survival. Since the present study only focuses on gender differences relating to the short term impact of business grants on firm performance, it is of interest for future work to examine effects in the longer term. New research on longer term effects may also provide more insights into how to design an optimal relief program to assist female business owners in the aftermath of disasters.

## Supporting information

S1 Fig Timeline for intervention and surveys of microenterprises in Sri Lanka (13 waves). (TIF)

S2 Fig Treatment assignment 1 (May 2005). (TIF)

S3 Fig Treatment assignment 2 (November 2005). (TIF)

S4 Fig Trends in real profit across 11 survey waves. (TIF)

S1 File Supporting evidence: This file includes Tables A to I in Supporting information in PDF. (PDF)

## References

[1] Hannah R, Roser M. Natural Disasters. Our World in Data; 2014. Available from: https://ourworldindata.org/natural-disasters

[2] Strömberg D. Natural Disasters, Economic Development, and Humanitarian Aid Journal of Economic Perspectives. 2007; 21(3):199–222. doi: 10.1257/jep.21.3.199

[3] Asgary A, Anjum MI, Azimi N. Disaster recovery and business continuity after the 2010 flood in Pakistan: Case of small businesses. International Journal of Disaster Risk Reduction. 2012; 2:46–56. doi: 10.1016/j.ijdrr.2012.08.001

[4] Ballesteros MM, Domingo SN. Building Philippine SMEs Resilience to Natural Disasters. PIDS Discussion Paper Series 2015–2020. 2015.

[5] Sarmiento JP, Hoberman G, Jerath M, Ferreira Jordao G. Disaster risk management and business education: the case of small and medium enterprises. AD-minister. 2016; 28:73–90. doi: 10.17230/ad-minister.28.4

[6] Nigg JM, Tierney K. Explaining Differential Outcomes in the Small Business Disaster Loan Application Process. Preliminary Paper No. 156. Disaster Research Center, University of Delaware, Newark, DE. 1990.

[7] Marshall MI, Niehm LS, Sydnor SB, Schrank HL. Predicting small business demise after a natural disaster: an analysis of pre-existing conditions. Natural Hazards. 2015; 79:331–354. doi: 10.1007/s11069-015-1845-0

[8] Gallagher J, Hartley D, Rohlin S. Weathering an Unexpected Financial Shock: The Role of Disaster Assistance on Household Finance and Business Survival. Journal of the Association of Environmental and Resource Economists. Forthcoming.

[9] Hanna B, Fisker P, Tarp F. Cash Grants To Manufacturers After Cyclone Idai: RCT Evidence From Mozambique. WIDER Working Paper 2021/87 Helsinki:UNU-WIDER, 2021.

[10] Banford A, Froude CK. Ecofeminism and Natural Disasters: Sri Lankan Women Post-Tsunami. Journal of International Women’s Studies. 2015; 16(2):170–187.

[11] De Mel S, McKenzie D, Woodruff C. Sri Lanka Microenterprise Survey (SLMS) 2005–2010. https://microdata.worldbank.org/index.php/catalog/1243. Dataset downloaded on: 2020–10–30.

[12] De Mel S, McKenzie D, Woodruff C. Returns to Capital in Microenterprises: Evidence from a Field Experiment. The Quarterly Journal of Economics. 2008; 123(4):1329–1372. doi: 10.1162/qjec.2008.123.4.1329

[13] De Mel S, McKenzie D, Woodruff C. Are Women More Credit Constrained? Experimental Evidence on Gender and Microenterprise Returns. American Economic Journal: Applied Economics. 2009; 1(3):1–32.

[14] De Mel S, McKenzie D, Woodruff C. Enterprise Recovery Following Natural Disasters. The Economic Journal. 2012; 122(559):64–91. doi: 10.1111/j.1468-0297.2011.02475.x

[15] Black N. Social Feminism. Ithaca, NY: Cornell University Press. 1989.

[16] Fischer EM, Reuber AR, Dyke LS. A theoretical overview and extension of research on sex, gender, and entrepreneurship. Journal of Business Venturing. 1993; 8(2):151–168. doi: 10.1016/0883-9026(93)90017-Y

[17] Gine X, Mansuri G. Money or Ideas? A Field Experiment on Constraints to Entrepreneurship in Rural Pakistan. World Bank Policy Research Working Paper No. 6959. 2014.

[18] Fafchamps M, McKenzie D, Quinn S, Woodruff C. Microenterprise growth and the flypaper effect: Evidence from a randomized experiment in Ghana. Journal of Development Economics. 2014; 106:211–226. doi: 10.1016/j.jdeveco.2013.09.010

[19] Fiala N. Returns to Microcredit, Cash Grants and Training for Male and Female Microentrepreneurs in Uganda. World Development. 2018; 105:189–200. doi: 10.1016/j.worlddev.2017.12.027

[20] Pellegrini G, Muccigrosso T. Do subsidized new firms survive longer? Evidence from a counterfactual approach. Regional Studies. 2016; 51(10):1483–1493. doi: 10.1080/00343404.2016.1190814

[21] Srhoj S, Škrinjarić B, Radas S. Closing the Finance Gap by Nudging: Impact Assessment of Public Grants for Women Entrepreneurs. EIZ Working Papers. 2019.

[22] Srhoj S, Škrinjarić B, Radas S. Bidding against the odds? The impact evaluation of grants for young micro and small firms during the recession. Small Business Economics. 2021; 56(1):83–103. doi: 10.1007/s11187-019-00200-6

[23] Haushofer J, Shapiro J. The Short-term Impact of Unconditional Cash Transfers to the Poor: Experimental Evidence from Kenya. The Quarterly Journal of Economics. 2016; 131(4):1973–2042. doi: 10.1093/qje/qjw025 33087990 PMC7575201

[24] Kilburn K, Thirumurthy H, Halpern CT, Pettifor A, Handa S. Effects of a large-scale unconditional cash transfer program on mental health outcomes of young people in Kenya. Journal of Adolescent Health. 2016; 58(2):223–229. doi: 10.1016/j.jadohealth.2015.09.023 26576822 PMC4724529

[25] Shangani S, Operario D, Genberg B, Kirwa K, Midoun M, Atwoli L, et al. Unconditional government cash transfers in support of orphaned and vulnerable adolescents in Western Kenya: is there an association with psychological wellbeing?. PLOS ONE. 2017; 12(5): e0178076. 10.1371/journal.pone.0178076 28562627 PMC5451046

[26] Angeles G, de Hoop J, Handa S, Kilburn K, Milazzo A, Peterman A. Government of Malawi’s unconditional cash transfer improves youth mental health. Social Science & Medicine. 2019; 225:108–19. 10.1016/j.socscimed.2019.01.037 30826585 PMC6829911

[27] Ohrnberger J, Anselmi L, Fichera E, Sutton M. The effect of cash transfers on mental health: Opening the black box—A study from South Africa. Social Science & Medicine. 2020; 260:113181. 10.1016/j.socscimed.2020.113181 32688162 PMC7441310

[28] Tozan Y, Sun S, Capasso A, Wang JSH, Neilands TB, Bahar OS, et al. Evaluation of a savings-led family-based economic empowerment intervention for AIDS-affected adolescents in Uganda: a four-year follow-up on efficacy and cost-effectiveness. PLOS ONE. 2019; 14(12): e0226809. 10.1371/journal.pone.0226809 31891601 PMC6938344

[29] Paxson C, Schady N. Does money matter? the effects of cash transfers on child health and cognitive development in rural Ecuador. Economic Development and Cultural Change. 2010; 59(1):187–229. doi: 10.1086/655458 20821896

[30] Özler B, Hallman K, Guimond MF, Kelvin EA, Rogers M, Karnley E. Girl Empower—A gender transformative mentoring and cash transfer intervention to promote adolescent well-being: Impact findings from a cluster-randomized controlled trial in Liberia. SSM-Population Health. 2020; 10:100527. 10.1016/j.ssmph.2019.100527 31890847 PMC6928354

[31] De Mel S, McKenzie D, Woodruff C. One-time transfers of cash or capital have long-lasting effects on microenterprises in Sri Lanka. Science. 2012; 335(6071):962–966. https://www.science.org/doi/abs/10.1126/science.1212973 22363007 10.1126/science.1212973

[32] Perera T. Creative Destruction and the Aftermath of the Tsunami: Aiding the Recovery of Southern Sri Lankan Small Businesses in the Face of Inertia. Industry and Higher Education. 2007; 21(1):99–107. 10.5367/000000007780222769

[33] World Vision. Sri Lanka Tsunami Response Final Report: December 2004–December 2007. World Vision. 2007.

[34] Carvalho VM, Nirei M, Saito YU, Tahbaz-Salehi A. Supply Chain Disruptions: Evidence from the Great East Japan Earthquake. The Quarterly Journal of Economics. 2021; 136(2):1255–1321. 10.1093/qje/qjaa044

[35] Croson R, Gneezy U. Gender Differences in Preferences. Journal of Economic Literature. 2009; 47(2):448–74. 10.1257/jel.47.2.448

[36] Eckel C, Grossman P. Differences in the Economic Decisions of Men and Women: Experimental Evidence. In Plott CR, Smith VL, editors, Handbook of Experimental Economics Results Volume 1. Amsterdam The Netherlands: Elsevier. 2008. pp.509–519.

[37] Dohmen T, Falk A, Huffman D, Sunde U, Schupp J, Wagner GG. Individual risk attitudes: Measurement, Determinants, and Behavioral consequences. Journal of the European Economic Association. 2011; 9(3):522–550. 10.1111/j.1542-4774.2011.01015.x

[38] Mueller G, Plug E. Estimating the Effect of Personality on Male and Female Earnings. ILR Review. 2006; 60(1):3–22. 10.1177/001979390606000101

[39] Qian N. Missing Women and the Price of Tea in China: The Effect of Sex-Specific Earnings on Sex Imbalance. The Quarterly Journal of Economics. 2008; 123(3):1251–1285. 10.1162/qjec.2008.123.3.1251

[40] Boboni GJ. Is the Allocation of Resources within the Household Efficient? New Evidence from a Randomized Experiment. Journal of Political Economy. 2009; 117(3):453–503. 10.1086/600076

[41] Plagerson S, Patel V, Harpham T, Kielmann K, Mathee A. Does money matter for mental health? Evidence from the Child Support Grants in Johannesburg, South Africa. Global Public Health. 2011; 6(7):760–776. 10.1080/17441692.2010.516267 20938853

[42] Wincent J, Ortqvist D, Drnovsek M. The entrepreneur’s role stressors and proclivity for a venture withdrawal. Scandinavian Journal of Management. 2008; 24(3):232–246. 10.1016/j.scaman.2008.04.001

[43] Hessels J, Rietveld CA, Thurik AR, Van der Zwan P. Depression and Entrepreneurial Exit. Academy of Management Perspectives. 2018; 32(3):323–339. 10.5465/amp.2016.0183

[44] Parida PK. Natural Disaster and Women’s Mental Health. Social Change. 2015; 45(2):256–275. 10.1177/0049085715574189

[45] Tatsuki S, Hayashi H, Yamori K, Noda T, Tamura K, Koshiyama K. Model construction and testing of psychological recovery processes from the Kobe earthquake disaster experiences I: life recovery process scale construction using the 2002 public restoration housing residents population survey data. Proceedings of the 3rd. Workshop for “Comparative Study on Urban Earthquake Disaster Management”. 2003; pp. 23–28.

[46] Pfarr C, Schmid A, Schneider U. Estimating Ordered Categorical Variables Using Panel Data: A Generalized Ordered Probit Model with an Autofit Procedure. Econometrics: Single Equation Models eJournal. 2010.

[47] Long JS. Regression models for categorical and limited dependent variables (Advanced Quantitative Techniques in the Social Sciences Series, Vol. 7). Thousand Oaks, CA: Sage Publications, Inc; 1997.

[48] IFRC. World Disasters Report 2020: Come Heat or High Water. International Federation of Red Cross and Red Crescent Societies. 2020. Available from: https://www.ifrc.org/document/world-disasters-report-2020

