# Supplementary figures and images for: Business grants following natural disasters and their different impact on the performance of female and male-owned microenterprises: Evidence from Sri Lanka
Source: PLoS One. 2022 Dec 21;17(12):e0279418. doi: 10.1371/journal.pone.0279418 (PMC9770362; doi:10.1371/journal.pone.0279418)

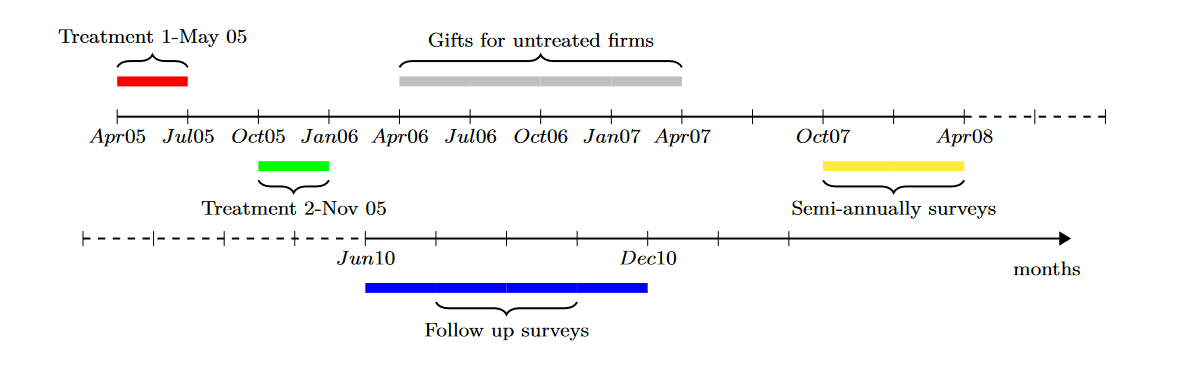

Supplement: S1 Fig — (TIF) [file pone.0279418.s001.tif]

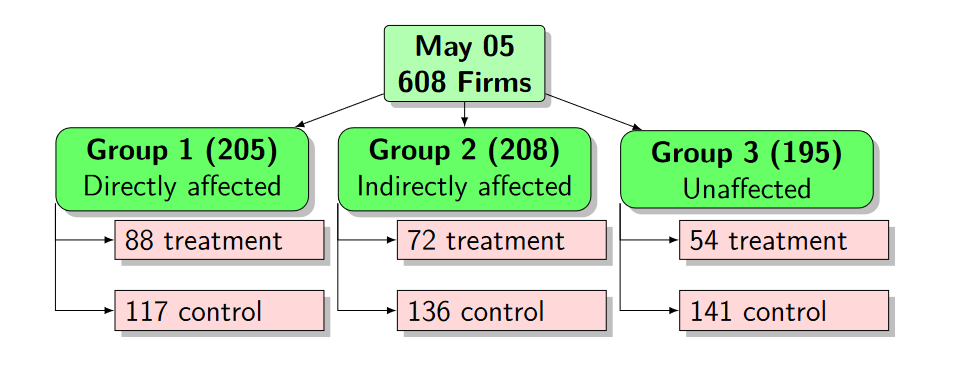

Supplement: S2 Fig — (TIF) [file pone.0279418.s002.tif]

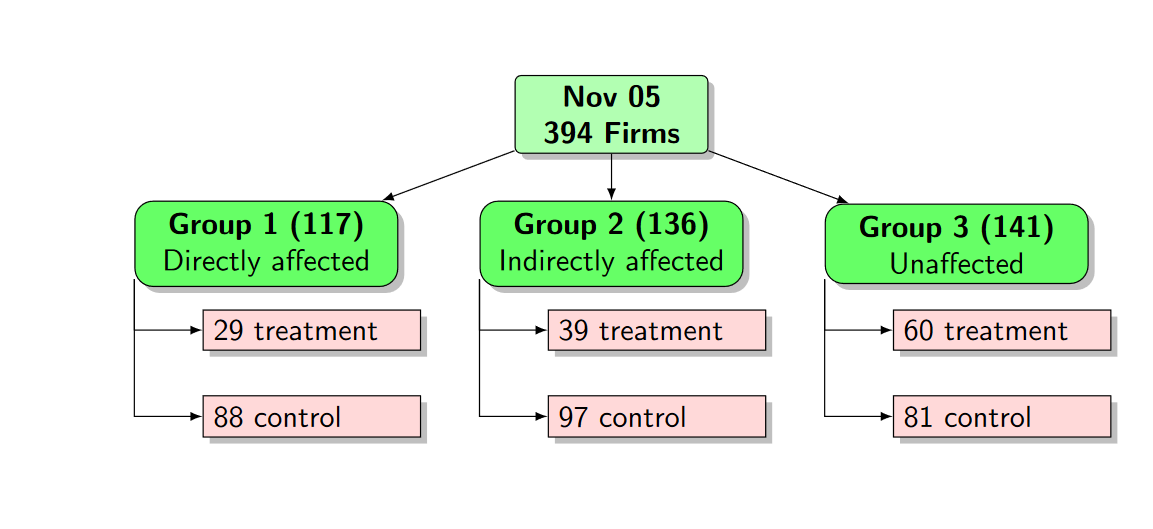

Supplement: S3 Fig — (TIF) [file pone.0279418.s003.tif]

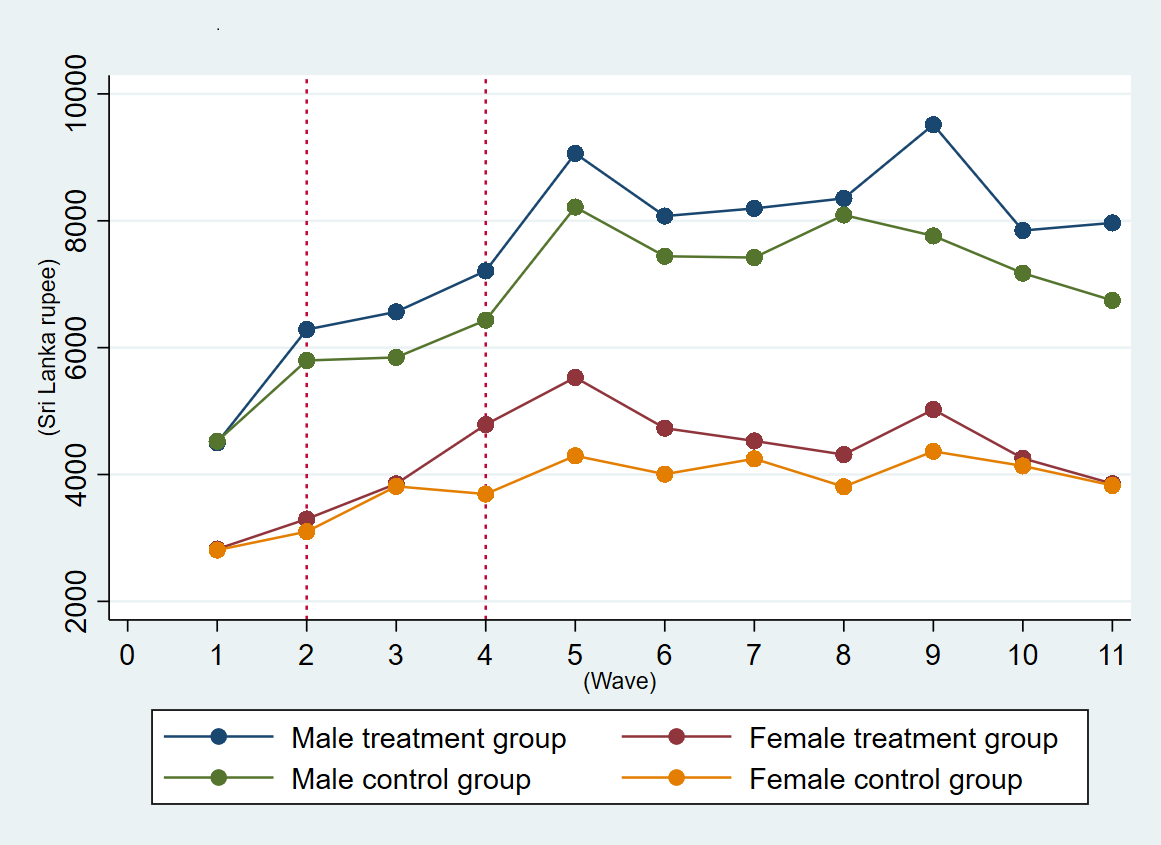

Supplement: S4 Fig — (TIF) [file pone.0279418.s004.tif]
