# Supplementary material for: Business grants following natural disasters and their different impact on the performance of female and male-owned microenterprises: Evidence from Sri Lanka
Source: PLoS One. 2022 Dec 21;17(12):e0279418. doi: 10.1371/journal.pone.0279418 (PMC9770362; doi:10.1371/journal.pone.0279418)
Supplement: S1 File — (PDF) [file pone.0279418.s005.pdf]

Online Supplemental Materials:
Business grants following natural disasters and their different impact on the performance of female and male-owned microenterprises: Evidence from Sri Lanka

Ha Luong [1,2,*]

1 Department of Economics, University of Barcelona, Barcelona, Spain
2 Barcelona Institute of Economics (IEB), Barcelona, Spain

¤Current Address: Department of Economics, University of Barcelona, Barcelona, Spain
***Email: ha.luong@ub.edu**

Table A to Table I in Supporting information

Table A: **Description of variables used in the study**

Variable	Definition
**Ability**	Measure the ability of owners by principal component analysis (time to solve a maze, years of education, self efficacy and digit-span recall score)
**Experience**	Equal 1 if the owner worked in the same sector before, and 0 otherwise
**Owner age**	The age of the owner at the baseline survey time
**Married**	Equal 1 if the owner is married, and 0 otherwise
**Female**	Equal 1 if the owner is female, and 0 otherwise
**Migrant**	Equal 1 if the owner is migrant, and 0 otherwise
**Father's education**	The highest level of education attained by the father of the owner (0 = no schooling, 1 = Year 1, 2 = Year 2, Years 3 - 11 code as 3 - 11, 12 = Year 12, 13 = Year 13, 14 = Some years of university, 15 = University undergraduate degree, 16 = Post-graduate university work, 17 = Technical college)
**Mother's education**	The highest level of education attained by the mother of the owner (0 = no schooling, 1 = Year 1, 2 = Year 2, Years 3 - 11 code as 3 - 11, 12 = Year 12, 13 = Year 13, 14 = Some years of university, 15 = University undergraduate degree, 16 = Post-graduate university work, 17 = Technical college)

Continued on next page

Table A: **Description of variables used in the study** (Continued)

Variable	Definition
**Household size**	Measure the number of people in the household of the owner
**Financial literacy**	Equal 1 if the owner answers correctly at least one question related to financial knowledge, and 0 otherwise
**Risk aversion**	Measure from a lottery exercise: the higher the value, the more risk averse
**Optimistic**	Measure from questions about the owner's imagination of their best possible life and worst possible life (a picture with 9 rungs, top=best, bottom=worst)
**Locus of control**	The sum of responses from three likert questions: *I plan tasks carefully*, *I made up my mind quickly* and *I save regularly* (1=Disagree strongly; 2=Disagree; 3=Neutral; 4=Agree 5=Agree strongly)
**Willingness to take risk**	Measure from the response of this question *Are you generally a person who is fully prepared to take risks or do you try to avoid taking risks? Please tick a box on the scale, where the value 0 means:"unwilling to take risks" and the value 10 means: "fully prepared to take risks"* in Wave 5
**Asset index**	The first principal component of 17 household assets
**Working hour**	Number of hours that the owner worked in the week before a survey wave
**Hit by tsunami**	Equal 1 if the owner was hit by water during the tsunami, and 0 otherwise
**Relatives dead**	Equal 1 if the owner has relatives killed in the tsunami, and 0 otherwise
**Pre-investment**	Equal 1 if the owner invested at least 5000 Sri Lankan rupees (LKR) when opening the business, and 0 otherwise
**Retail/trade**	Equal 1 if the firm belongs to retail or trade sectors, and 0 if the firm belongs to manufacturing or service sectors
**Firm age**	The time between the establishment year of a firm and the baseline survey (in years)
**Total number of workers**	Number of workers in the firm, including wage or salaried workers, partners and unpaid workers
**Asset damage**	Equal 1 if the firm has business asset damaged or destroyed by the tsunami, and 0 otherwise
**Real profit**	Business income in the last month before the survey wave after deducting all expenses including the wages of employees, but not including any income that the owners paid themselves in LKR (deflated to April 2005)
**Real sales**	Firm sales of the last month before the survey wave in LKR (deflated to April 2005)

Continued on next page

Table A: **Description of variables used in the study** (Continued)

Variable	Definition
**Capital stock**	Monthly firm capital stock without land, including equipment and inventories minus equipment rent in LKR (deflated to April 2005)
**Inputs purchase**	Monthly raw material expenditure for manufacturing firms and items for resale for retail and trade and service firms in LKR (deflated to April 2005)
**Interest payment**	Monthly interest paid on loans, which is a category of business expenses in LKR (deflated to April 2005)
**Close**	Equal 1 if the owner changed their line of business/changed both their line of business and their location/was no longer self employed/was not engaged in business activity, and 0 otherwise
**Time**	Number of months between the baseline time and the time when the owner closed their initial business
**Basic needs**	Monthly expenditure that includes food consumption (the expenditure on groceries, food consumed at home and food consumed outside the home), housing (house rent, taxes, maintenance, water bill), healthcare and clothing in LKR (deflated to April 2005)
**Education**	Monthly expenditure that includes school supplies, school fees and donations in LKR (deflated to April 2005)

Table B. Testing the different treatment effects across four types of treatment

	(1)	(2)
	**Real profit**	**Real sales**
Cash 10000	2123.0*** (821.2)	6115.2** (2575.9)
Cash 20000	536.9 (747.7)	1546.0 (3623.0)
In-kind 10000	909.7* (500.8)	3067.7 (3485.6)
In-kind 20000	1279.0 (805.1)	5498.0 (3917.9)
Firm FE	✓	✓
Wave FE	✓	✓
Testing the differences in treatment (p-value):		
Cash 10000 vs. Cash 20000	0.1438	0.2932
Cash 10000 vs. In-kind 10000	0.1926	0.4714
Cash 10000 vs. In-kind 20000	0.4538	0.8932
Cash 20000 vs. In-kind 10000	0.6675	0.7582
Cash 20000 vs. In-kind 20000	0.4891	0.4520
In-kind 10000 vs. In-kind 20000	0.6874	0.6375
Observations	5427	5505
Number of clusters	601	602
R^2	0.031	0.017

Note: Standard errors, clustered at the enterprise level, are shown in parentheses. Cash 10000 equals 1 if a firm received 10,000 LKR in cash, and 0 otherwise. In-kind 10000 equals 1 if a firm received 10,000 LKR in-kind, and 0 otherwise. Cash 20000 equals 1 if a firm received 20,000 LKR in cash, and 0 otherwise. In-kind 20000 equals 1 if a firm received 20,000 LKR in-kind, and 0 otherwise. $^*p < 0.10$, $^{**}p < 0.05$, $^{***}p < 0.01$.

Table C. Treatment effects on real sales of female-owned firms and male-owned firms

	Dependent variable is real sales					
	(1)	(2)	(3)	(4)	(5)	(6)
Treatment	5230.1** (2590.6)	4757.2* (2487.1)	985.8 (3585.7)	5152.2** (2553.7)	2465.0 (2334.6)	-2300.3 (3524.7)
Treatment×Female	-3782.5 (3115.0)	-3360.1 (2982.4)	-3288.8 (3082.9)	-3793.5 (3125.9)	-3009.8 (3039.0)	-2054.4 (2863.8)
Treatment×Risk aversion		1025.1 (1033.4)				1071.7 (1035.0)
Treatment×Married			4863.7 (3386.1)			4654.7 (3303.7)
Treatment×Asset Index				308.6 (1034.0)		162.2 (1002.4)
Treatment × Retail/trade					4974.1* (2611.0)	5276.4** (2676.3)
Observations	5505	5505	5505	5505	5505	5505
Number of clusters	602	602	602	602	602	602
R^2	0.017	0.017	0.017	0.017	0.018	0.019

Notes: Standard errors, clustered at the enterprise level, are shown in parentheses. Risk aversion is measured from a lottery game played with real money by each entrepreneur in wave 2. Married is a dummy variable that equals 1 if the owner is married, and 0 otherwise. Asset index is the first principal component of 17 household assets. Retail/trade is a dummy variable that equals 1 if the firm belongs to the retail or trade sectors and 0 if the firm belongs to the manufacturing or service sectors. *$p < 0.10$, **$p < 0.05$, ***$p < 0.01$.

Table D. Randomization check by gender (10,000 LKR treatment versus control)

	Male			Female		
Variable	Mean control	Mean treat	P-val. diff	Mean control	Mean treat	P-val. diff
Owner characteristics						
Ability	-0.071	-0.129	(0.721)	0.010	0.104	(0.565)
Experience	0.695	0.604	(0.164)	0.639	0.620	(0.767)
Age	42.370	43.890	(0.312)	41.030	42.086	(0.457)
Married	0.815	0.870	(0.258)	0.748	0.836	(0.089)*
Migrant	0.148	0.100	(0.276)	0.119	0.155	(0.400)
Working hour	0.593	0.620	(0.673)	0.385	0.310	(0.217)
Household size	4.948	5.170	(0.348)	4.867	4.802	(0.769)
Firm characteristics						
Pre-investment	0.637	0.660	(0.717)	0.459	0.483	(0.711)
Retail/Trade	0.407	0.540	(0.044)**	0.333	0.328	(0.924)
Firm age	10.17	14.34	(0.005)***	10.17	11.21	(0.432)
Real profit	4,523	4,682	(0.769)	2,813	2,744	(0.832)
Real sales	14,036	14,748	(0.757)	8,802	8,147	(0.657)
Invested capital without land	32,472	29,829	(0.516)	21,524	23,940	(0.444)
Total number of workers	1.356	1.470	(0.178)	1.407	1.448	(0.606)
Interest payment	50.000	21.500	(0.399)	136.874	90.517	(0.558)
Observations	135	100		135	116	

Note: *$p < 0.10$, **$p < 0.05$, ***$p < 0.01$.

Table E. Randomization check by gender (20,000 LKR treatment versus control)

	Male			Female		
Variable	Mean control	Mean treat	P-val. diff	Mean control	Mean treat	P-val. diff
Owner characteristics						
Ability	-0.071	0.193	(0.156)	0.010	-0.152	(0.458)
Experience	0.695	0.714	(0.782)	0.639	0.512	(0.151)
Age	42.370	40.737	(0.303)	41.030	40.870	(0.936)
Married	0.815	0.895	(0.126)	0.748	0.674	(0.330)
Migrant	0.148	0.079	(0.143)	0.119	0.130	(0.832)
Working hour	0.593	0.592	(0.995)	0.385	0.565	(0.033)**
Household size	4.948	4.974	(0.915)	4.867	4.913	(0.873)
Firm characteristics						
Pre-investment	0.637	0.592	(0.521)	0.459	0.543	(0.326)
Retail/trade	0.407	0.461	(0.456)	0.333	0.413	(0.331)
Firm age	10.17	9.17	(0.469)	10.17	8.97	(0.506)
Real profit	4,523	4,262	(0.632)	2,813	3,001	(0.726)
Real sales	14,036	13,617	(0.862)	8,802	9,534	(0.714)
Invested capital without land	32,472	31,138	(0.752)	21,524	25,402	(0.351)
Total number of workers	1.356	1.461	(0.232)	1.407	1.478	(0.495)
Interest payment	50.000	288.684	(0.014)**	136.874	61.957	(0.507)
Observations	135	76		135	46	

Note: *$p < 0.10$, **$p < 0.05$, ***$p < 0.01$.

Table F. Treatment effect on firms with business asset damage caused by the tsunami

	**Dependent variable is real profit**	
	Female-owned firms	Male-owned firms
Treatment	2142.4** (1015.4)	2947.0* (1641.9)
Firm FE	✓	✓
Wave FE	✓	✓
Observations	1054	936
Number of clusters	115	106
R^2	0.036	0.042

Standard errors, clustered at the enterprise level, are shown in parentheses.
*$p < 0.10$, **$p < 0.05$, ***$p < 0.01$.

Table G. Weekly working hours in the male sub-samples by two treatment amounts

	**Dependent variable is weekly working hours**	
	(1)	(2)
Treatment 10000	6.785** (3.186)	
Treatment 20000		-0.494 (3.709)
Firm FE	✓	✓
Wave FE	✓	✓
Observations	1774	1587
Number of clusters	189	168
R^2	0.010	0.011

Standard errors, clustered at the owner level, are shown in parentheses.
*$p < 0.10$, **$p < 0.05$, ***$p < 0.01$.

Table H. Survival analysis result when the hazard rate is allowed to change over time (Weibull distribution)

	Dependent variable is time	
	(1)	(2)
Female	-0.459<br>(0.341)	-0.435<br>(0.342)
Ever Treatment 10000	-0.497<br>(0.359)	
Ever Treatment 10000 × Female	1.245***<br>(0.475)	
Ever Treatment 20000		-0.560<br>(0.402)
Ever Treatment 20000 × Female		0.841<br>(0.583)
Controls	✓	✓
Ln(p)	-0.131<br>(0.094)	-0.216*<br>(0.120)
Observations	470	376

Note: Robust standard errors are shown in parentheses. The parameter p is the shape parameter (define the shape of Weibull distribution). In Column (1), the estimate for ln(p) is -0.131 and not statistically significant, which means that the Weibull model is not a better "fit" than the exponential model. In Column (2), the estimate for ln(p) is -0.216 and statistically significant, which means that the hazards are decreasing monotonically over time. However, the result is very similar when the paper applies the exponential model. *$p < 0.10$, ** $p < 0.05$, ***$p < 0.01$.

Table I. The difference in initial business closures between female and male owners (whole sample)

	**Logit**	**LPM**	**Survival analysis**
	Close	Close	Time
	(1)	(2)	(3)
Female	-0.446<br>(0.361)	-0.0542<br>(0.0439)	-0.457<br>(0.343)
Ever Treatment	-0.435<br>(0.329)	-0.0568<br>(0.0433)	-0.521*<br>(0.312)
Ever Treatment × Female	1.166**<br>(0.468)	0.154**<br>(0.0604)	1.158***<br>(0.434)
Controls	✓	✓	✓
Observations	590	590	589

Note: Robust standard errors are shown in parentheses. Ever Treatment equals 1 if the owner ever received the grant from the experiment, and 0 otherwise. In the survival analysis, the result indicates that the treatment has a positive impact on the survival of male-owned firms. The negative coefficient of *Ever Treatment* implies that treated male owners have lower hazard than control male owners to close their firms. *$p < 0.10$, **$p < 0.05$, ***$p < 0.01$.
